# Identification of Extracellular DNA-Binding Proteins in the Biofilm Matrix

**DOI:** 10.1128/mBio.01137-19

**Published:** 2019-06-25

**Authors:** Jeffrey S. Kavanaugh, Caralyn E. Flack, Jessica Lister, Erica B. Ricker, Carolyn B. Ibberson, Christian Jenul, Derek E. Moormeier, Elizabeth A. Delmain, Kenneth W. Bayles, Alexander R. Horswill

**Affiliations:** a Department of Immunology and Microbiology, University of Colorado School of Medicine, Aurora, Colorado, USA; b Department of Microbiology, University of Iowa Carver College of Medicine, Iowa City, Iowa, USA; c Biology Department, University of Utah, Salt Lake City, Utah, USA; d Department of Microbiology and Pathology, University of Nebraska Medical Center, Omaha, Nebraska, USA; e Department of Veterans Affairs Eastern Colorado Healthcare System, Aurora, Colorado, USA; Harvard University

**Keywords:** MRSA, Southwestern blotting, *Staphylococcus aureus*, biofilms, eDNA, extracellular DNA, nuclease

## Abstract

We developed a new approach that couples Southwestern blotting and mass spectrometry to discover proteins that bind extracellular DNA (eDNA) in bacterial biofilms. Using Staphylococcus aureus as a model pathogen, we identified proteins with known DNA-binding activity and uncovered a series of lipoproteins with previously unrecognized DNA-binding activity. We demonstrated that expression of these lipoproteins results in an eDNA-dependent biofilm enhancement. Additionally, we found that while deletion of lipoproteins had a minimal impact on biofilm accumulation, these lipoprotein mutations increased biofilm porosity, suggesting that lipoproteins and their associated interactions contribute to biofilm structure. For one of the lipoproteins, SaeP, we showed that the biofilm phenotype requires the lipoprotein to be anchored to the outside of the cellular membrane, and we further showed that increased SaeP expression correlates with more retention of high-molecular-weight DNA on the bacterial cell surface. SaeP is a known auxiliary protein of the SaeRS system, and we also demonstrated that the levels of SaeP correlate with nuclease production, which can further impact biofilm development. It has been reported that S. aureus biofilms are stabilized by positively charged cytoplasmic proteins that are released into the extracellular environment, where they make favorable electrostatic interactions with the negatively charged cell surface and eDNA. In this work we extend this electrostatic net model to include secreted eDNA-binding proteins and membrane-attached lipoproteins that can function as anchor points between eDNA in the biofilm matrix and the bacterial cell surface.

## Abstract (precis)

Many bacteria are capable of forming biofilms encased in a matrix of self-produced extracellular polymeric substances (EPS) that protects them from chemotherapies and the host defenses. As a result of these inherent resistance mechanisms, bacterial biofilms are extremely difficult to eradicate and are associated with chronic wounds, orthopedic and surgical wound infections, and invasive infections, such as infective endocarditis and osteomyelitis. It is therefore important to understand the nature of the interactions between the bacterial cell surface and EPS that stabilize biofilms. Extracellular DNA (eDNA) has been recognized as an EPS constituent for many bacterial species and has been shown to be important in promoting biofilm formation. Using Staphylococcus aureus biofilms, we show that membrane-attached lipoproteins can interact with the eDNA in the biofilm matrix and promote biofilm formation, which suggests that lipoproteins are potential targets for novel therapies aimed at disrupting bacterial biofilms.

## INTRODUCTION

The capacity to form a biofilm is of fundamental importance in the ability of bacteria to cause chronic wounds, to infect indwelling medical devices, and to cause severe, invasive diseases, such as osteomyelitis and endocarditis. The opportunistic pathogen Staphylococcus aureus is particularly adept at forming biofilms, making it the most commonly identified bacterial species in chronic wounds (1–3), the leading cause of infective endocarditis (4, 5), the second most common causative agent in periprosthetic orthopedic infections (6), and the most common cause of osteomyelitis (7). Once attached to an implanted device or host tissue and growing within the extracellular matrix (ECM) of a biofilm, S. aureus suppresses the host immune response (8, 9) and becomes highly resistant to antibiotic chemotherapy (10), allowing for persistence within the host. Given the recalcitrant nature of biofilm-associated infections, interventions that target the biofilm matrix could offer therapeutic benefits (11). As such, the exact nature of the biofilm matrix and the mechanisms by which individual bacteria interact with and attach to matrix material are topics of recent interest.

The bacterial biofilm matrix, which typically accounts for 90% or more of the biofilm dry weight, is crucial for maintaining hydration and the structural integrity of biofilms (12, 13). Our understanding of what constitutes the ECM has evolved over decades, such that it is now generally believed to consist of self-produced extracellular polymeric substances (EPS) that fall within three primary categories: (i) exopolysaccharide, (ii) extracellular and cell surface-associated proteins/adhesins, and (iii) extracellular DNA (eDNA). The relative abundance of the macromolecules that fall within these three classes of matrix constituents can vary depending on the bacterial species and the conditions, such as the medium composition or shear stress (12, 13), under which the biofilm was grown. In the case of S. aureus, our understanding of the contributions that the classes of EPS components make to biofilm formation and maintenance is continually evolving, with the number and variety of macromolecules that fall within the scope of EPS ever increasing.

The exopolysaccharide consisting of poly-β(1-6)-*N*-acetylglucosamine (PNAG), also referred to as polysaccharide intercellular adhesin (PIA) (14, 15), was the first component of S. aureus EPS to be identified. Recent atomic force microscopy studies (16) indicate that positively charged PIA promotes cell-cell adhesion between S. aureus cells via multivalent electrostatic interactions with polyanionic teichoic acids and lipoteichoic acids. Initially, PIA was thought to be essential for S. aureus biofilm formation, but more recently, an increasing number of reports (reviewed in reference 14) have demonstrated that biofilms can be made by strains lacking the *ica* locus, responsible for PIA synthesis. The biofilms described in these reports are sensitive to proteases and dependent on the presence of various cell wall-attached (CWA) proteins. Subsequently, extensive and ongoing studies have shown that CWA proteins (reviewed in references 17 and 18) contribute to biofilm development by promoting the attachment of S. aureus to host cells and tissues through specific interactions with host proteins, as well as through self-association between individual cells.

Recent studies suggest that extracellular DNA (eDNA) may play a more significant role than microbial surface components recognizing adhesive matrix molecules (MSCRAMM) adhesin proteins when S. aureus forms biofilms on abiotic surfaces (19). Using a microfluidic flow cell system, Moormeier and colleagues (19) found that mutant strains that were deficient in the production of MSCRAMM proteins, including ﬁbronectin-binding proteins A and B (FnbA and FnbB) (20), extracellular matrix and plasma binding protein (Empbp) (21), clumping factors A and B (ClfA and ClfB) (22, 23), protein A (24), elastin-binding protein (EbpS) (25), Sas family proteins (26, 27), and serine-aspartate repeat (Sdr) family proteins (28, 29), grew biofilms that were indistinguishable from those grown by the wild type (WT). Likewise, biofilm formation was not altered in mutants deficient in PIA synthesis, consistent with the previous finding that S. aureus strains of the USA300 lineage form an *ica*-independent biofilm (30). In contrast, biofilm formation was reduced in the *atlA* mutant that lacks the murine hydrolase AtlA, which had previously been shown to promote biofilm formation by functioning as the autolysin responsible for the release of eDNA that incorporates into the matrix (31). Additionally, Moormeier et al. identified a new phase of biofilm development, termed “exodus,” that is dependent on the SaeRS regulatory system and staphylococcal nuclease (Nuc) activity, which again suggests that eDNA plays a crucial role in early biofilm maturation (19, 32). It was also observed that the addition of exogenous Nuc disrupted biofilm formation, but only if exogenous nuclease was introduced during the exodus phase, raising the possibility that eDNA may be protected from digestion by eDNA-binding proteins in early developmental stages. Since deletion of MSCRAMM proteins failed to impact biofilm development, the eDNA-binding proteins may belong to a protein class other than the MSCRAMM proteins.

Recent reports from the Losick group suggest that recycled cytoplasmic proteins may “moonlight” as biofilm matrix proteins that bind eDNA in the S. aureus biofilm (33, 34), potentially protecting the eDNA from digestion by Nuc or host DNase I. By disrupting biofilms and suspending cells at various pHs, these cytoplasmic proteins were found to be associated with the outside of cells through electrostatic interactions (35). Treatment of biofilm cells with either proteinase K or DNase severely reduced the ability of bacteria to form clumps, indicating that both proteins and eDNA contribute to maintaining biofilm integrity (34). Importantly, addition of exogenous DNA to DNase-treated cells, but not proteinase K-treated cells, restored clumping, highlighting the importance of eDNA in forming interconnections between cells within the biofilm. The authors proposed an electrostatic net model for the role of eDNA in biofilm maintenance, in which negatively charged eDNA interacts with positively charged cytoplasmic proteins released during cell autolysis. These cytoplasmic proteins in turn interact with the negatively charged cell surface molecules, such as phospholipids and teichoic acids. The model has recently been bolstered by a comprehensive mass spectrophotometric analysis of the S. aureus intracellular, biofilm ECM, and biofilm flowthrough proteomes (35), which found that the average pI of proteins within the biofilm ECM was over pH 8 and significantly greater than the average pI of proteins found in either the intracellular or flowthrough proteomes. Like the Losick group (33, 34), Graf and colleagues (35) found that the ECM contained cytoplasmic proteins in abundance, but they also found that the ECM was highly populated with secreted virulence factors and ribosomal proteins.

Here we report the results of a Southwestern (SW) blotting screen for eDNA-binding proteins that complements and extends the electrostatic net model for S. aureus biofilms. A combination of SW blotting and mass spectrometry (MS) approaches was used to probe membranes for proteins with eDNA-binding activity. Proteins previously known to possess DNA-binding activity (such as IsaB, Atl, and Eap) were identified, and more importantly, previously unrecognized DNA-binding activity was discovered for a number of membrane-associated proteins and membrane-anchored lipoproteins. We also show that overexpression of identified eDNA-binding proteins results in increased retention of surface eDNA, which correlates with enhanced biofilm biomass. For one of the lipoproteins, SaeP, it was further demonstrated that the ability to enhance biofilm formation was dependent on the protein being localized and attached to the extracellular face of the cell membrane. Bioinformatic analysis identified additional lipoproteins with high pIs and potential DNA-binding activity, including lipoproteins belonging to the conserved staphylococcal antigens (Csa) family. Importantly, deletion of the Csa proteins increased the porosity of the biofilm, suggesting that membrane-attached lipoproteins contribute to biofilm tortuosity. Collectively, our findings indicate that eDNA-binding lipoproteins represent a previously unrecognized contributor to S. aureus biofilm formation by linking individual bacterial cells together through noncovalent cross-links with high-molecular-weight (high-MW) eDNA found within the matrix. For SaeP, it was further demonstrated that the ability to enhance biofilm formation was partly dependent on the inhibition of Nuc production, consistent with the role of SaeP in modulating the activity of the SaeRS two-component system.

## RESULTS

### Identification of potential eDNA-binding proteins.

**FIG 1:**
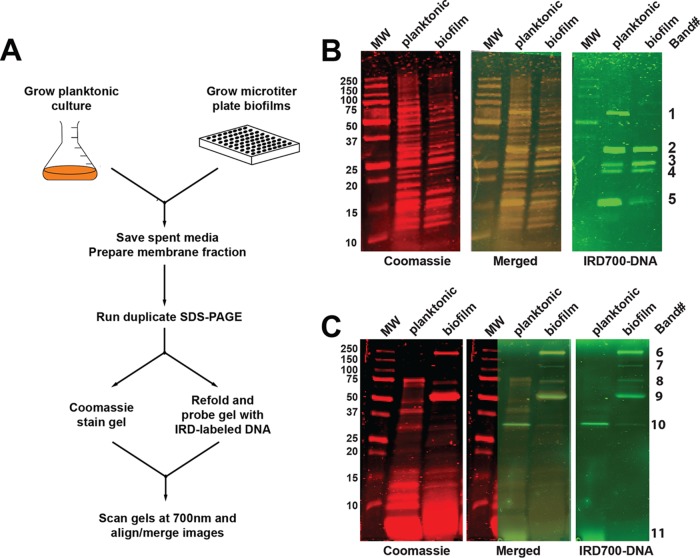
SW blotting approach. (A) Schematic of the SW blotting experimental design. (B) SW analysis of membrane-associated proteins prepared from planktonic and biofilm bacteria. The left panel, with red bands, is an image of the 700-nm scan of a Coomassie-stained gel, and the far-right panel, with green bands, is an image of the 700-nm scan of a duplicate gel taken after the proteins were renatured and the gel was probed with IRD700-labeled DNA. In the center panel, the IRD700-labeled DNA panel was overlaid on the Coomassie-stained panel. Labels for the MW markers (in kilodaltons) are shown at the far left, and identification band numbers for the bands that were excised and sent for MS analysis are shown at the far right. (C) SW analysis of soluble proteins in medium from planktonic cultures and biofilms.

To better understand the composition of the S. aureus biofilm matrix, we developed a screen based on Southwestern (SW) blotting techniques, which are classically used to characterize protein-DNA interactions. We adapted this technique to identify proteins isolated from the biofilm matrix with nonspecific DNA-binding activity, as proteins with sequence-specific DNA binding would be less likely to play a major biofilm structural role. To carry out this experiment, the USA300 strain LAC was grown in planktonic and biofilm cultures overnight (Fig. 1A). Cells were collected from these cultures and fractionated to yield cellular membranes, since proteins associated with the membrane fraction could potentially provide bridging interactions between the cell surface and matrix components, such as eDNA. The cell membrane fraction was run on duplicate nonreducing SDS-PAGE gels, and one gel was Coomassie stained for protein band visualization (Fig. 1B, left). The remaining gel was treated with a Triton X-100-based buffer to allow for protein refolding within the gel and then incubated with an IRD700-labeled DNA probe (Fig. 1B, right). The gel images were then overlaid (Fig. 1B, center) to identify bands on the Coomassie-stained gel with DNA-binding activity. The same procedure was applied to the media from the planktonic and biofilm cultures (Fig. 1C) in order to compare the results with those of membrane SW blotting, as we reasoned that the media could potentially contain important eDNA-binding proteins. In total, 11 bands (labeled bands 1 through 11 along the right edge of Fig. 1B and C) were excised and sent for identification by MS analysis.

While the SW analysis utilized purified membranes, both the abbreviated (Table 1) and complete (see Table S1 in the supplemental material) lists of the top protein identifications returned by MS analysis included membrane proteins, cell wall-associated proteins, secreted proteins, as well as cytoplasmic proteins, highlighting the fact that it is not possible to completely resolve subcellular fractions from one another. Presumably, the nonmembrane proteins that were identified are the ones with the highest binding affinity for membrane components, which could include phospholipids, membrane-attached or embedded proteins, and/or lipoteichoic acids, since these proteins pelleted with membranes, while other proteins did not. Alternatively, the nonmembrane proteins could have associated with eDNA, which in turn associated with components of the membrane fraction. Of note, with the exception of band 6 (analysis failed to yield an identification), numerous proteins for each band were identified as being present with 100% certainty. This presents a challenge in terms of assigning responsibility for the IRD700-DNA bands to any particular protein or set of proteins, which will be addressed in more detail below. Overall, there was very good agreement between the list in Table S1 and the results of the proteome analysis recently published by Graf et al. (35), as indicated by the positions of the proteins within the rank order of abundance in the ECM. Of significance, the majority of the membrane fraction proteins in the list were found by Graf et al. (35) to have high fold changes when comparing their abundance in ECM relative to that in the biofilm flowthrough (see the FC [fold change] ECM/FT [flowthrough] column in Table S1), meaning that once they were outside the cells, these proteins tended to remain associated with the biofilm rather than to be washed away in the flowthrough. Since the SW analysis-identified proteins are potential DNA-binding proteins, it suggests that these proteins may remain associated with the biofilm through interactions with eDNA. The list of proteins in Table S1 is dominated by highly alkaline proteins, with 41 of the proteins having pIs of 9 or greater and another 8 having pIs of between 8 and 9, meaning that roughly 70% of the proteins in the list would be expected to carry a net positive charge at the acidic pH that is typical of biofilms. It is therefore reasonable to expect them to make favorable electrostatic interactions with negatively charged eDNA. Ribosomal proteins, many of which are known to directly interact with rRNA and/or tRNA, are highly represented, constituting roughly 30% of the list, in excellent agreement with the ECM proteome characterized by Graf et al. (35). Four of the ribosomal proteins and an additional two nonribosomal cytoplasmic proteins in the list (see the Foulston et al. column in Table S1) were previously identified as being part of the biofilm matrix by the studies that led to the development of the electrostatic net model (33, 34), indicating that the proteins identified by the SW technique also corroborated approximately 20% of the cytoplasmic proteins identified in this study. Importantly, a number of proteins known to bind DNA were identified, including Atl (bands 1, 8, and 9) (36), SarA (band 5) (37), IsaB (band 5) (38), Eap (band 1) (39), and the phenol-soluble modulins (PSMs; band 11) (40). Interestingly, in the case of PSMs, it was further shown that the binding of PSMs to DNA protected the DNA from digestion by DNase (40), and in the case of Eap, it was found that this protein can condense extended DNA strands (39), raising the possibility that Eap might also afford protection from host DNase.

**TABLE 1:** Representative hits from SW analysis screen

Protein source and band	ORF	Name	Location	Mol wt (kDa)	pI
Membrane fraction					
1	SAUSA300_1917	Eap, extracellular adherence protein	Secreted membrane associated	65.7	9.9
1	SAUSA300_0955	AtlA amidase domain	Surface, equatorial ring	60.8	9.6
1	SAUSA300_1193	Aerobic glycerol-3- phosphate dehydrogenase	Cytoplasmic	62.4	7.0
2	SAUSA300_1790	PrsA peptidyl-prolyl *cis*-*trans* isomerase	Membrane lipoprotein	35.6	9.0
2	SAUSA300_0618	ABC transporter, Mn/Zn transporter substrate-binding protein	Membrane protein	34.7	8.7
2	SAUSA300_0419	Lpl lipoprotein	Membrane lipoprotein	31.4	8.8
3	SAUSA300_2201	RplA, 50S ribosomal protein L1	Cytoplasmic/ribosome	30.1	10.7
4	SAUSA300_2142	Asp23	Cytoplasmic	20.3	8.2
4	SAUSA300_2187	RpsE, 30S ribosomal protein S5	Cytoplasmic/ribosome	17.7	9.9
4	SAUSA300_0079	CopL lipoprotein	Membrane lipoprotein	20.1	9.0
5	SAUSA300_0605	SarA	Cytoplasmic	14.7	8.1
5	SAUSA300_0693	SaeP	Membrane lipoprotein	16.0	9.1
5	SAUSA300_2573	IsaB	Cytoplasmic	19.4	9.7
Spent medium					
7	SAUSA300_0955	AtlA, full length	Surface, equatorial ring	116.5	9.6
8	SAUSA300_0320	Geh, triacylglycerol lipase	Secreted	72.2	9.0
8	SAUSA300_0955	AtlA amidase domain	Surface equatorial ring	60.8	9.6
8	SAUSA300_2579	*N*-Acetylmuramoyl-l- alanine amidase	Surface, equatorial ring	69.2	6.4
9	SAUSA300_0703	LtaS, SpsB-liberated domain	Surface, equatorial ring	49.3	8.6
9	SAUSA300_0955	AtlA glucosaminidase domain	Surface, equatorial ring	54.4	9.7
10	SAUSA300_1058	Hla, alpha-toxin	Secreted	36.0	8.7
10	SAUSA300_1150	Elongation factor Ts	Cytoplasmic	32.5	5.1
10	SAUSA300_0553	Elongation factor Tu	Cytoplasmic	43.1	4.5
10	SAUSA300_0235	Lactate dehydrogenase	Cytoplasmic	29.4	5.1
11	SAUSA300_1988	α-Toxin	Secreted	3.0	8.7
11	SAUSA300_	PSM α4	Secreted	2.2	9.7
11	SAUSA300_1067	PSM β1	Secreted	4.5	4.9
11	SAUSA300_	PSM α1	Secreted	2.3	9.7

10.1128/mBio.01137-19.7 TABLE S1 Comprehensive list of hits from the SW analysis screen. Download Table S1, DOCX file, 0.04 MB. Copyright © 2019 Kavanaugh et al. 2019 Kavanaugh et al. This content is distributed under the terms of the Creative Commons Attribution 4.0 International license.

### Confirmation of eDNA-binding proteins via Southwestern analysis.

Given that the MS analysis indicated the presence of multiple proteins within any given SW blotting band, follow-up studies were required to determine which protein or proteins possessed DNA-binding activity. These studies consisted of running SW analysis on a strain containing a deletion of the target gene or a strain in which the target gene was expressed at an elevated level from a plasmid, and in some cases, DNA-binding activity was confirmed using purified protein in electromobility shift assays (EMSAs). The exact combination of techniques utilized varied for each protein to be tested.

**FIG 2:**
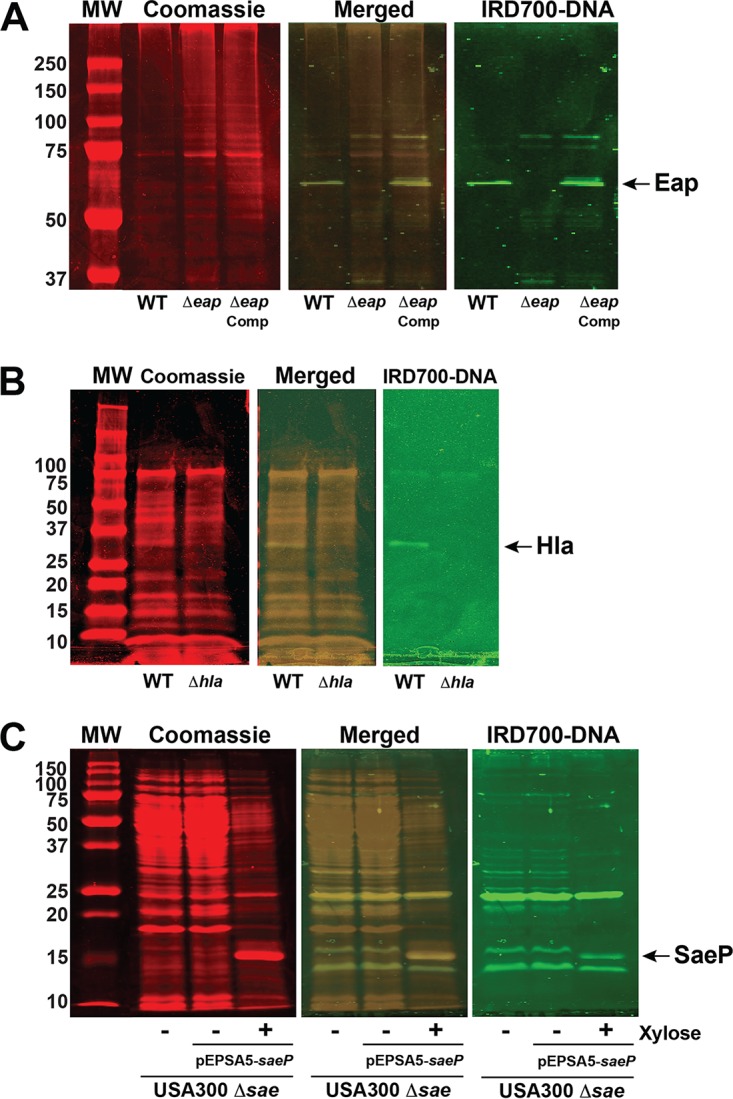
Confirmation of SW band identifications. (A) SW analysis of membranes prepared from planktonic cultures of the Newman WT, Δ*eap* mutant, and complemented (Comp) Δ*eap* mutant strains. The position of the Eap band is indicated by the arrow at the far right. (B) SW analysis of medium from planktonic cultures of the LAC WT and Δ*hla* mutant strains. The position of the Hla band is indicated by the arrow at the far right. (C) SW analysis of membranes prepared from biofilms of the Δ*sae* mutant of LAC and the Δ*sae* mutant of LAC containing pEPSA5 expressing *saeP*. When expression of *saeP* is induced with xylose, a new band (indicated by the arrow at the far right) corresponding to SaeP is evident in both the Coomassie-stained and IRD700-labeled-DNA-probed gel. The numbers to the left of the gels are MW (in kilodaltons).

In the case of SW blotting band 1 (Table 1), MS analysis identified three potential DNA-binding proteins: the amidase domain of AtlA, Eap, and aerobic glycerol-3-phosphatre dehydrogenase. Since our MS results (Table S1) indicated a high abundance for Eap, we reasoned that at least some of the IRD700-DNA fluorescence was due to probe binding to Eap. However, since the amidase domain of AtlA is known to bind DNA (36), it seemed unlikely that SW analysis of an Δ*eap* mutant of strain LAC would work as a means of confirming DNA-binding activity for Eap. However, given that Eap is known to be expressed at a very high level in strain Newman (41), we reasoned that SW analysis of a knockout mutant would work in this genetic background. SW analysis (Fig. 2A) was conducted using membranes prepared from the Newman wild-type, Δ*eap*, and complemented Δ*eap* strains (42), and a labeled band was obvious at the appropriate molecular weight (MW) in the wild-type and complemented strain lanes, while it was absent from the Δ*eap* mutant lane. This observation confirmed that Eap contributes, at least in part, to SW blotting band 1 seen in Fig. 1B. Subsequently, the DNA-binding activity of Eap has been confirmed by others using EMSAs (39).

SW analyses of knockout mutants of strain LAC were also conducted to confirm the potential DNA-binding proteins in bands 7, 8, and 10 (Table 1). In the case of alpha-toxin (Hla) in SW analysis band 10, the labeled band was clearly absent (Fig. 2B) in the SW analysis of medium from a planktonic culture of a Δ*hla* mutant (43), which is consistent with reports that Hla is incorporated into S. aureus biofilms and binds eDNA (44) and the identification of Hla within both the biofilm ECM and the flowthrough (35) but skewed toward the flowthrough. In the cases of membranes prepared from a Δ*atlA* mutant grown under biofilm conditions, SW analysis bands 7 and 8, it was not possible to confirm the protein identity, owing primarily to the weak intensity of these bands in the wild-type SW analysis. The candidate eDNA-binding proteins in SW analysis bands 2, 3, 4, 5, and 9 were not amenable to validation of knockout mutants owing to the fact that they (bands 2 through 5) contained at least one essential ribosomal protein (45) or the candidate protein itself is essential, as was the case for LtaS (band 9). Since band 5 contains two proteins, SarA and IsaB, that are already known to bind DNA, an alternative SW analysis approach was used to confirm the DNA-binding activity of the third candidate identified in this band, the SaeP lipoprotein. SaeP was expressed in an *saePQRS* deletion mutant (46) under the control of a xylose-inducible promoter on plasmid pEPSA5 (45). As shown in Fig. 2C, there was a new labeled band in the xylose-induced lane that was absent in both the *sae* mutant and uninduced lanes, consistent with SaeP-binding DNA.

### SW analysis-identified lipoproteins bind DNA.

**FIG 3:**
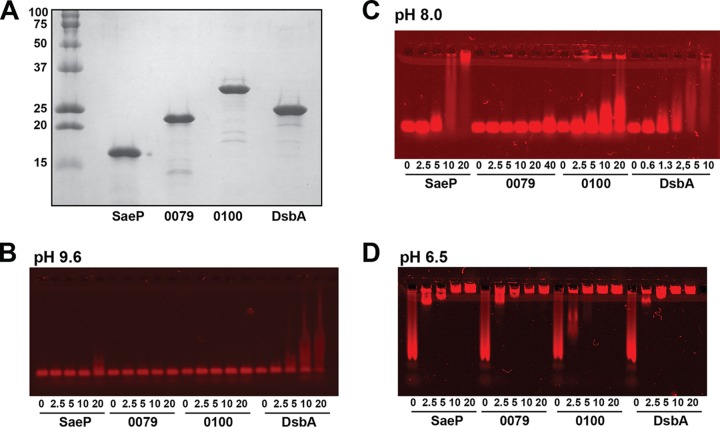
DNA-binding activity of purified lipoproteins SaeP, 0079 (CopL), 0100, and DsbA. (A) SDS-PAGE of Ni-affinity chromatography-purified His-tagged lipoproteins that were cloned in pET28a and expressed in E. coli. The numbers to the left of the gel are MW (in kilodaltons). (B to D) EMSAs run at pH 9.6 (B), pH 8.0 (C), and pH 6.7 (D), using ethanolamine-Capso, Tris-acetate, and bis-Tris–Aces pK_a_-matched buffer systems, respectively. For each protein, a serial 2-fold dilution series (20 to 2.5 μM or 40 to 2.5 μM) was combined with a 259-bp, IRD700-labeled DNA probe at a final concentration of 5 nM, and the mixture was incubated for 30 min at room temperature and then electrophoresed on 1% agarose gels that had been cast with the appropriate pK_a_-matched buffer. Following electrophoresis, gels were scanned using the 700-nm channel on an Odyssey CLx imager (LI-COR, Omaha, NE) and visualized using Image Studio software.

With the identification of SaeP and other lipoproteins in the SW analysis screen, EMSAs were used to directly test the DNA binding of four purified lipoproteins. These included SaeP and 0079, which has recently been identified to be copper-binding protein CopL (47), from the SW analysis screen (Table 1), and two lipoproteins chosen at random, open reading frame (ORF) 0100, which belongs to the same protein family as 0419 identified in the SW analysis screen (see “Bioinformatics analysis and perspectives of potential eDNA-binding proteins” below), and DsbA, a known thiol-disulfide oxidoreductase protein which was included as a control (48). Soluble, His-tagged versions of all four lipoproteins lacking the secretion leader were cloned, overexpressed in Escherichia coli, and purified to homogeneity by Ni-affinity chromatography (Fig. 3A). When initial EMSAs were run using low-percentage polyacrylamide gels, the IRD700-labeled double-stranded DNA (dsDNA) probe failed to migrate out of the wells in lanes containing protein (data not shown). This observation suggests that high-MW protein-DNA complexes formed as a result of nonspecific binding to the probe. In order to resolve the high-MW complexes and to determine whether electrostatic interactions drive nonspecific binding to the DNA, the proteins were dialyzed into buffer systems of various pHs (pH 9.6, 8.0, and 6.5) and agarose gels were used in place of polyacrylamide gels (Fig. 3B to D). The subsequent EMSAs clearly indicated that all four lipoproteins bound the dsDNA probe with affinities that were in part dependent on the overall net charges of the proteins. At pH 9.6, all four proteins are expected to carry net negative charges, given the pIs of 9.2, 9.0, 7.7, and 9.2 for SaeP, 0079, 0100, and DsbA, respectively, and very little shifting of the probe was observed (Fig. 3B). At pH 8.0, the predicted protein net charges ranged from −1 to +7, and both SaeP and DsbA showed pronounced binding (Fig. 3C), while 0100 showed some binding and 0079 did not bind at all. At pH 6.5, the proteins were quite positively charged, with net charges ranging from +4 to +14, and each of the four showed nearly complete probe binding (Fig. 3D). Based on the apparent relationship between the net protein charge and affinity for DNA, all of the proteins should have even higher affinities for DNA at the acidic pHs associated with S. aureus biofilm formation (20).

### Contribution of lipoproteins to S. aureus biofilm formation.

Considering the identification of the SaeP lipoprotein in the SW analysis screen (Fig. 2C and Table 1), the confirmation of its DNA-binding activity by EMSA (Fig. 3), and the connection between Sae regulation and biofilm formation (49, 50), we became interested in determining the contribution of SaeP to S. aureus biofilm development. A markerless knockout of *saeP* was constructed in the USA300 LAC strain and engineered in a way as to not impact the expression of the *saeQRS* transcript. Growth curves revealed no changes in growth for the Δ*saeP* mutant (Fig. S1A), and, surprisingly, microtiter assays showed no change in biofilm formation in the Δ*saeP* mutant from that in the WT (Fig. S1B). If SaeP promotes biofilm formation through interactions with eDNA in the biofilm matrix, then failure of the Δ*saeP* mutation to impact biofilm formation may be due the presence of lipoproteins with redundant DNA-binding activity. Given this, we decided to pursue an alternative strategy and investigate whether increasing the expression level of SaeP could impact biofilm formation. Since the SW and EMSA analyses identified additional lipoproteins with DNA-binding activity, we chose to examine multiple lipoproteins at the same time.

10.1128/mBio.01137-19.1 FIG S1 Deletion of S. aureus *saeP* does not impact growth or biofilm formation. (A) Growth curve of biofilms of the WT, Δ*saeP*, and Δ*saePQRS* strains. Bacteria were subcultured 1:100 in TSB and grown in a 37°C shaker, and the OD_600_ was measured on a plate reader. (B) Comparison of biofilms of the WT, Δ*saeP*, and Δ*saePQRS* strains. Microtiter biofilms were grown in TSB plus 0.4% glucose in 48-well plates. Plates were incubated in a 37°C humidified shaker at 500 rpm for 24 h before the adherent biomass was stained with 0.1% crystal violet. Error bars indicate the standard deviation for four replicate wells. Download FIG S1, TIF file, 0.4 MB. Copyright © 2019 Kavanaugh et al. 2019 Kavanaugh et al. This content is distributed under the terms of the Creative Commons Attribution 4.0 International license.

**FIG 4:**
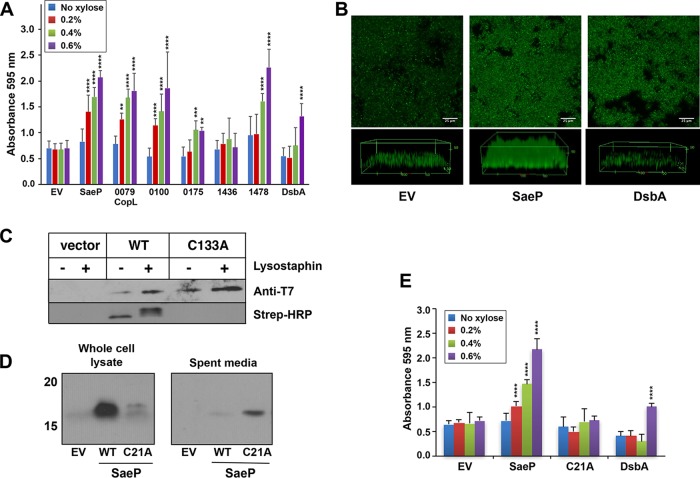
Impact of lipoprotein expression and localization on S. aureus biofilm development. (A) LAC strains were constructed with the pEPSA5 empty vector (EV) or various lipoproteins. Biofilms were grown in 48-well plates in TSB plus 0.4% glucose and xylose at a range of concentrations (0 to 0.6%) to induce protein expression. Error bars are the standard deviation for 12 wells (three experiments with four wells per lipoprotein). Statistics for two-way analysis of variance are *P* values. **, *P* ≤ 0.005; ***, *P* ≤ 0.0005; ****, *P* ≤ 0.00005. (B) Confocal microscope images of flow cell biofilms for the strain with the empty vector (left), SaeP-expressing strains (center), and DsbA-expressing strains (right). Biofilms were grown for 2.5 days in 2% TSB supplemented with 0.2% glucose, 1 μg/ml of chloramphenicol, and 0.1% xylose. (C) SCAM analysis of native SaeP in strain LAC shows that cysteine residue C133 is modified without lysostaphin pretreatment, indicating that SaeP is located on the cell surface. Protein expression was evaluated by T7 immunoblotting, while cysteine labeling was analyzed using Strep-HRP. (D) Western blot analyses performed with whole-cell lysates and bacterial supernatants of LAC expressing WT SaeP-T7 or the C21A mutant of SaeP-T7 show that the WT protein is associated with cells, whereas the C21A mutant is found in the supernatant, confirming that the C21A mutant is no longer a lipoprotein. (E) Biofilm assays, performed as described in the legend to panel A, of WT and C21A mutation-expressing strains (compared to strains expressing DsbA as a control) show that the mutant no longer supports enhanced biofilm accumulation. Error bars are the standard deviation for three replicate wells. Statistics for two-way analysis of variance are *P* values. ****, *P* ≤ 0.00005.

For initial testing, the four lipoproteins with EMSA-confirmed DNA-binding activity (SaeP, 0079 [CopL], 0100, and DsbA) and three others with various pIs (USA300 ORFs 0175 with a pI of 8.7, 1436 with a pI of 6.2, and 1478 with a pI of 6.8) were chosen from the chromosome for comparison. Each lipoprotein was cloned into pEPSA5 to allow xylose induction and then expressed at various levels in USA300 strain LAC. As shown in Fig. 4A, a number of the lipoproteins had a significant impact on S. aureus biofilm formation compared to the effect of the empty-vector control. Notably, SaeP and 0079 (CopL), as well as 0100, all dramatically enhanced the biofilm formation capacity, even at lower xylose levels. 1478 also dramatically enhanced the biofilm formation capacity at high xylose levels but had a more modest enhancement at lower xylose levels. Likewise, DsbA enhanced biofilm formation only at high levels of xylose. Similarly, 0175 exhibited a very small increase in biofilm formation only at the higher xylose concentrations, and 1436, the protein with the lowest pI, had no impact on biofilm formation.

As a further measure of the impact of overexpressing the lipoproteins upon biofilm formation, two of the strains overexpressing the lipoproteins SaeP and DsbA were grown in flow cells. Consistent with the results of the microtiter plate biofilm assays, biofilm enhancement was also observed when SaeP was expressed under flow conditions (Fig. 4B), where the basal expression of SaeP expression from the pEPSA5 vector resulted in an increase in the average biomass compared to that for the empty-vector control (51.90 versus 22.61 μm^3^/μm^2^), and an intermediate level of biofilm formation was observed with DsbA. The finding that multiple lipoproteins with confirmed DNA-binding activity can dramatically enhance biofilm formation suggests that under normal growth conditions, multiple lipoproteins, including some yet to be identified or tested, may contribute to biofilm stability by cross-linking noncovalently with eDNA in the biofilm matrix.

### Evaluating the impact of SaeP localization on biofilm enhancement.

As shown above, SaeP and other lipoproteins identified in the SW analysis screen (Table 1) bind DNA (Fig. 3B to D) and enhance biofilm formation (Fig. 4A and B), suggesting that they may represent an extension of the electrostatic net model of S. aureus biofilm formation. In this regard, the noncovalent, electrostatic interactions between lipoproteins and the high-MW eDNA might function as direct cross-links between the bacterial surface and the biofilm matrix, thus promoting bacterial aggregation as well as biofilm formation and stabilization. If the lipoproteins function through this mechanism, then the enhancement should be dependent upon proper anchoring to the membrane, the accessibility of the DNA-binding domain, and the presence of eDNA.

We first investigated the impact of SaeP localization on enhancement of biofilm formation. Previous work showed that SaeP can be detected in the spent medium of an *lgt* mutant (51), supporting its assignment as a lipoprotein. To verify that SaeP is exposed to the extracellular environment, we used the substituted cysteine accessibility method (SCAM) (52), which relies on a thiol-reactive reagent, *N*-(3-maleimidylpropionyl)biocytin (MPB), to attach biotin to cysteine side chains. Since MPB does not penetrate S. aureus, only cysteines on the outside of the cell are labeled, unless the bacteria are pretreated with lysostaphin, in which case intracellular cysteines also are labeled. Application of SCAM requires that the protein being analyzed contain only one reactive cysteine; hence, the method often requires that cysteine residues be added or removed from the protein through site-directed mutagenesis. However, given that SaeP contains only two cysteine residues, C21 and C133, C133 should be the only reactive cysteine in the mature lipoprotein, since C21 is the site of lipidation. Therefore, it was possible to conduct the SCAM analysis without modifying the native SaeP amino acid sequence. When S. aureus cells expressing SaeP (with a C-terminal T7 tag) were reacted with MPB, biotin-labeled C133 was detected in both the presence and the absence of lysostaphin pretreatment, indicating that C133 is exposed to the extracellular environment and that the domain is accessible (Fig. 4C). Interestingly, pretreatment with lysostaphin revealed three discrete bands labeled with MPB (streptavidin [Strep]-horseradish peroxidase [HRP] gel) migrating very close together, suggesting that the pre-SaeP, pro-SaeP, and mature SaeP forms were labeled, while the absence of lysostaphin pretreatment showed MPB labeling of only the fastest-migrating species, corresponding to the mature form of SaeP (53). MPB labeling of SaeP did not occur when C133 was mutated to an alanine, confirming that C133 is the reactive cysteine in SaeP.

In order to test whether anchoring of SaeP to the cellular membrane is necessary for biofilm enhancement, we generated a construct that eliminated the lipidation site (C21A). The C21A mutant was detected in culture supernatants, and very little was found in whole cells (Fig. 4D), suggesting that the C21A mutant is secreted and no longer a lipoprotein. Conversely, WT SaeP was detected predominantly in whole cells, as would be expected for a lipoprotein. Importantly, when the microtiter plate biofilm assay was repeated with various levels of WT SaeP or C21A mutant expression (compared to the level of expression of the DsbA control), WT SaeP caused a dramatic enhancement of biofilm formation, as expected, while the C21A mutant showed no increase in biofilm formation (Fig. 4E), indicating that membrane anchoring and surface localization are required for enhanced biofilm formation. As a final localization experiment, SaeP was purified by affinity chromatography and added directly to LAC biofilms at the time of culture inoculation. Over a wide range of SaeP concentrations, no changes in biofilm formation were evident (Fig. S2). Taken together, these results show that SaeP must be tethered to the S. aureus membrane surface on the extracellular face to mediate an increase in biofilm formation.

10.1128/mBio.01137-19.2 FIG S2 Exogenous SaeP addition does not impact S. aureus biofilms. Soluble, purified His-SaeP protein (from 0 to 5 mM) was added at time zero to LAC biofilms. Error bars are the standard deviation for four replicate wells. Download FIG S2, TIF file, 0.6 MB. Copyright © 2019 Kavanaugh et al. 2019 Kavanaugh et al. This content is distributed under the terms of the Creative Commons Attribution 4.0 International license.

### SaeP enhances biofilm capacity in an eDNA-dependent manner.

**FIG 5:**
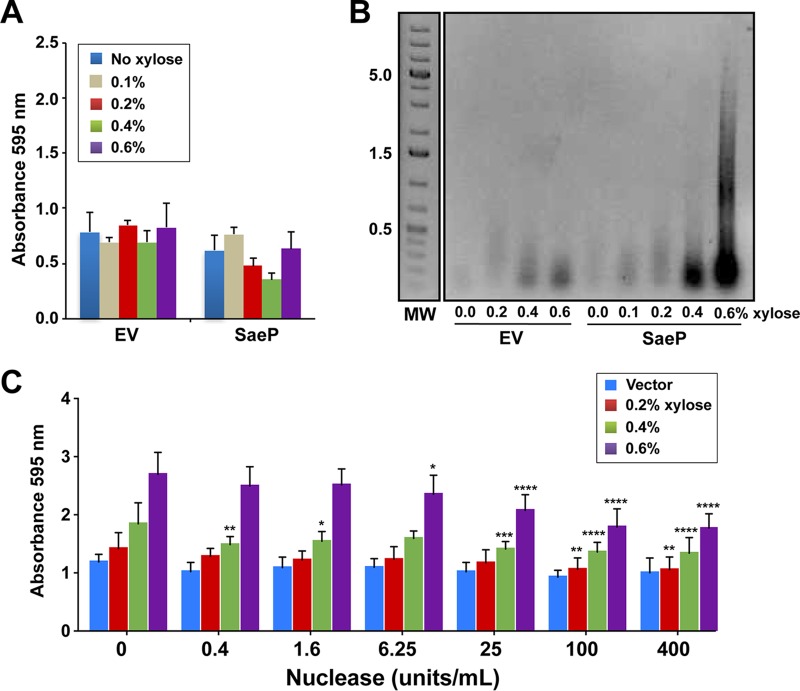
SaeP-induced biofilm enhancement is dependent on eDNA. (A) SaeP was expressed from pEPSA5 in an *atlA* mutant under biofilm-forming conditions using a range of xylose concentrations (0 to 0.6%) to induce protein expression. Error bars are the standard deviation for three replicate wells. (B) SaeP expression results in the accumulation of eDNA in the biofilm matrix. Static biofilms expressing either SaeP or DsbA were grown in TSB supplemented with 0.4% glucose and xylose at the indicated concentrations. eDNA isolated from the biofilm matrix was run on agarose gels. The numbers to the left of the gel are MW (in kilodaltons). (C) Biofilm enhancement of LAC expressing WT SaeP at 0.6% xylose is reduced when purified Nuc is added at time zero. Statistics for two-way analysis of variance are *P* values. *, *P* ≤ 0.05; **, *P* ≤ 0.005; ***, *P* ≤ 0.0005; ****, *P* ≤ 0.00005.

Considering the results presented above, we predicted that the SaeP-based biofilm enhancement would be mediated through interactions with eDNA. We tested this prediction using an *atlA* mutant, which has reduced autolysis and eDNA levels (54). Expression of *saeP* in the *atlA* mutant failed to enhance biofilm formation and resulted in a biofilm profile similar to that of the empty-vector control (Fig. 5A), suggesting a dependence on eDNA. To determine whether eDNA levels were altered by the presence of SaeP, biofilm matrix-associated eDNA was isolated from WT LAC cultures expressing SaeP. Substantially more high-MW eDNA than was observed for the empty-vector control was detected in the matrix at increased levels of SaeP expression (Fig. 5B); the empty-vector control had only a small amount of low-MW (<100-bp) DNA (Fig. 5B).

Since the presence of SaeP results in biofilms with higher eDNA levels, we predicted that the SaeP-dependent enhancement of biofilm formation should be sensitive to the presence of a nuclease. To test this prediction, a dilution series of purified S. aureus nuclease (Nuc) was added at the time of inoculation to cultures in which SaeP was expressed at high levels by xylose induction. At added Nuc concentrations of 6.5 U/ml and above, there was a significant, dose-dependent decrease in biofilm formation (Fig. 5C), further demonstrating the importance of eDNA to the SaeP-dependent biofilm enhancement.

### Contribution of lipoproteins to S. aureus biofilm structure.

Our collective results indicated that different membrane-attached lipoproteins likely contribute to the stability of S. aureus biofilms, suggesting that deletion of any one lipoprotein would not have a measurable impact on the amount of biofilm biomass. This was supported by our findings with the *saeP* mutant (Fig. S1B). Nevertheless, we reasoned that if a sufficient number of lipoproteins were inactivated, then the ability of S. aureus to form biofilms or the structure of the biofilm itself could be impacted. In this regard, the lipoproteins belonging to the conserved staphylococcal antigens (Csa) family presented a unique opportunity to test this hypothesis. As described in “Bioinformatics analysis and perspectives of potential eDNA-binding proteins” below, the Csa family in S. aureus USA300 strains consists of 14 highly homologous lipoproteins that are distributed within four distinct loci on the chromosome, and some of these lipoproteins, such as SAUSA300_0419, were identified in the SW analysis screen (Table 1). We tested the contribution of Csa lipoproteins to the biofilm structure by making serial deletions on the USA300 chromosome: Δ*locus I* and Δ*lpl* (also called locus III) single mutants, a Δ*locus I* Δ*lpl* double mutant, and a Δ*locus I* Δ*lpl* Δ*locus IV* triple mutant. The USA300 chromosome does not contain locus II.

**FIG 6:**
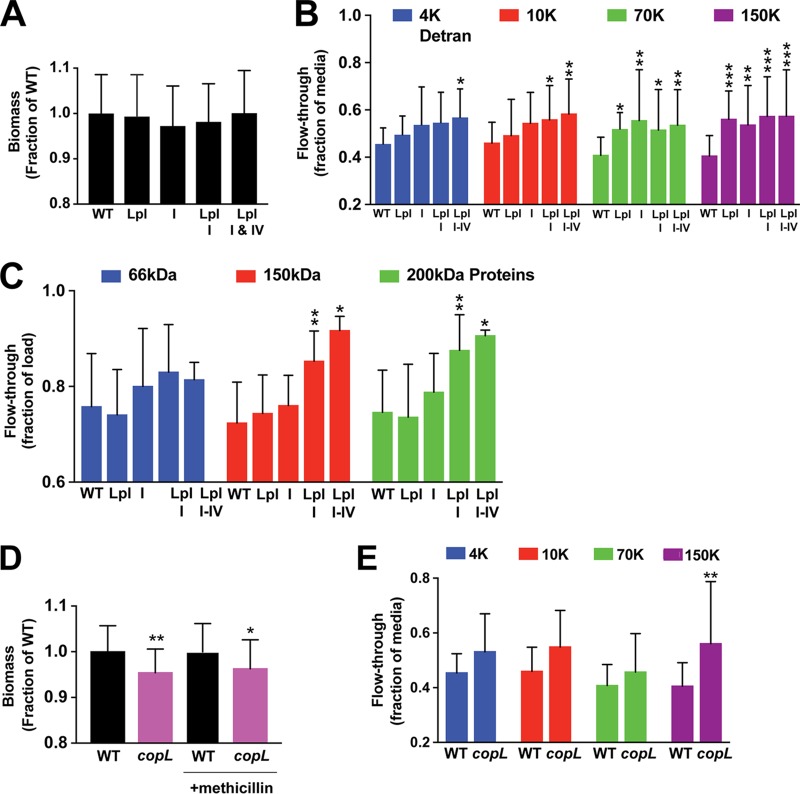
Lipoprotein mutants show increased biofilm porosity. (A) Biofilm biomass for Csa knockout mutants measured by crystal violet staining and expressed as a fraction of the WT biomass. Values are averages from either two or three experiments (>45 wells per strain tested). (B) Concentrations of different-molecular-weight FITC-dextrans in the flowthrough of filter-grown biofilms (12 wells per experiment). Concentrations are expressed as the fraction of the FITC-dextran concentration in the flowthrough of wells containing sterile medium (i.e., no-biofilm control wells). Statistics for two-way analysis of variance are *P* values. *, *P* ≤ 0.05; **, *P* ≤ 0.005; ***, *P* ≤ 0.0005. (C) Protein band intensities from SDS-PAGE of the filter-grown biofilm flowthrough, expressed as the fraction of the intensity for the corresponding band in the protein loading solution. Values are the averages from two experiments with >14 wells per strain tested (except that 3 wells were used for the triple mutant). Statistics for two-way analysis of variance are *P* values. *, *P* ≤ 0.05; **, *P* ≤ 0.005. (D) Biofilm biomass for the WT and CopL mutant measured by crystal violet staining and expressed as a fraction of the WT biomass. Values are averages from two experiments with >40 wells per strain tested. Statistics for one-way analysis of variance are *P* values. *, *P* ≤ 0.05; **, *P* ≤ 0.005. (E) Concentrations of different-molecular-weight FITC-dextrans in the flowthrough of filter-grown biofilms. Concentrations are expressed as the fraction of the FITC-dextran concentration in the flowthrough of wells containing sterile medium. Values are the averages from two experiments with 12 wells per strain per experiment. Statistics for two-way analysis of variance are *P* values. **, *P* ≤ 0.005.

Since it has been shown that sub-MICs of β-lactam antibiotics cause S. aureus to form thicker eDNA-dependent biofilms (55, 56), we used this protocol to initially assess biofilm formation by the Δ*csa* mutants. However, none of the Δ*csa* locus mutants showed a reduced biofilm formation capacity in this assay (Fig. 6A). Since S. aureus biofilms grown in the presence of sub-MICs were also found to be less porous than biofilms grown without antibiotic (55), we decided to test whether biofilm porosity was impacted in the Δ*csa* mutants. For this approach, we measured the movement of various-molecular-weight fluorescein isothiocyanate (FITC)-dextrans and proteins through biofilms that were grown on ultrafiltration membrane filters (Fig. 6A). Previous studies demonstrated that convection through biofilms grown on ultrafiltration membranes was impacted by the polysaccharides and/or eDNA in the biofilm (55, 57). Biofilms were grown on 96-well filter plates containing polyvinylidene difluoride (PVDF) membranes with 0.45-μm pores. After 24 h of static incubation at 37°C, culture supernatants were removed from the plates by aspiration and replaced with buffer containing FITC-dextran of a particular molecular weight (either 4,000 [4K], 10K, 70K, or 150K at 1 mg/ml) or a mixture of proteins of different molecular weights (bovine serum albumin [BSA] at 66 kDa, Saccharomyces cerevisiae yeast alcohol dehydrogenase [ADH] at 150 kDa, and β-amylase at 200 kDa). Filter plates were then subjected to a brief, low-speed centrifugation, and the concentrations of the FITC-dextran and proteins in the flowthrough were determined by fluorescence or densitometry of the SDS-PAGE gels, respectively.

As shown in Fig. 6B, serial deletion of the Csa lipoproteins resulted in an increased flowthrough of FITC-dextrans. Of note, as progressively more Csa loci were deleted, there was a trend toward increased passage for each FITC-dextran. However, the point at which a statistically significant increase in FITC-dextran flowthrough was observed was dependent on the number of Csa loci deleted. For the smallest 4K FITC-dextran, there was no significant increase in flowthrough until all three USA300 Csa loci were deleted. For the 10K FITC-dextran, there was a significant increase in flowthrough in both the triple and double deletion mutants, and for the 70K and 150K FITC-dextrans, there were significant increases in flowthrough for all the deletion mutants. This trend is consistent with the loss of interactions between the Csa lipoproteins on the bacterial cell surface and eDNA leading to larger pores and channels within the biofilm and, consequently, easier passage of the higher-molecular-weight FITC-dextrans through the biofilm. Importantly, the flowthrough of proteins (Fig. 6C) showed similar relationships between the number of loci deleted, the solute molecular weight, and flowthrough, reinforcing the interpretation of increased FITC-dextran flowthrough.

In order to test the generality of the inferred relationship between biofilm porosity and the number of interactions between the lipoproteins on the cell surface and the eDNA in the matrix, we repeated the analysis of FITC-dextran flowthrough using biofilms of a CopL (SAUSA300_0079) deletion mutant. Importantly, CopL was identified in the SW analysis screen (Table 1, band 4); purified CopL bound DNA, as indicated by EMSA analysis (Fig. 3); and overexpression of CopL resulted in increased biofilm biomass (Fig. 4A). Unlike the Δ*csa* mutants, the *copL* mutant did show a small but statistically significant decrease in biofilm biomass in both the absence and the presence of sub-MICs of antibiotic (Fig. 6D). When the FITC-dextran flowthrough was quantified (Fig. 6E), all the FITC-dextrans showed a trend toward increased rates of flowthrough in the *copL* mutant biofilms, which was consistent with the interpretation of the Δ*csa* mutant flowthrough measurements.

### Contribution of proteins Eap and IsaB identified by SW analysis to eDNA binding.

Besides the lipoprotein candidates, the SW blotting approach identified a number of other eDNA-binding proteins of interest (Table 1). We examined two of the better-characterized proteins identified in more detail, starting with the extracellular adherence protein (Eap) and immunodominant surface protein B (IsaB). Both Eap and IsaB are characterized, secreted proteins, and both are known to bind DNA (38, 39). Coupled with our SW analysis results (Fig. 2A), we were confident about their localization and eDNA-binding properties. To test the hypothesis that these proteins contribute to the structure of the S. aureus biofilm matrix, we first constructed knockouts of *eap* and *isaB* in the USA300 LAC background, and like the Δ*saeP* mutant and the Δ*csa* mutants, these mutants had no significant impact on biofilm formation in microtiter assays (Fig. S4). We decided to test the effect of these genes on biofilm formation in another strain background, HG001, where high levels of secreted IsaB were reported (58). In HG001, a 2-fold decrease in biofilm formation was observed in the *isaB* single mutant, as well as in a double mutant in which the *eap* gene was also disrupted (Fig. S4), but still no effect was observed for the *eap* mutant. We reasoned that Eap and IsaB might redundantly bind surface eDNA in the matrix, and their respective contributions may relate more to the biofilm structure than to a net contribution to biomass.

10.1128/mBio.01137-19.4 FIG S4 Impact of the Δ*isa* and Δ*eap* mutations, as well as the Δ*isa* Δ*eap* double mutation, on microtiter plate biofilm formation in the WT LAC (blue bars) and HG001 (light gray bars) genetic backgrounds. *P* values were determined by Student’s *t* test. ****, *P* ≤ 0.0005. Download FIG S4, TIF file, 0.7 MB. Copyright © 2019 Kavanaugh et al. 2019 Kavanaugh et al. This content is distributed under the terms of the Creative Commons Attribution 4.0 International license.

**FIG 7:**
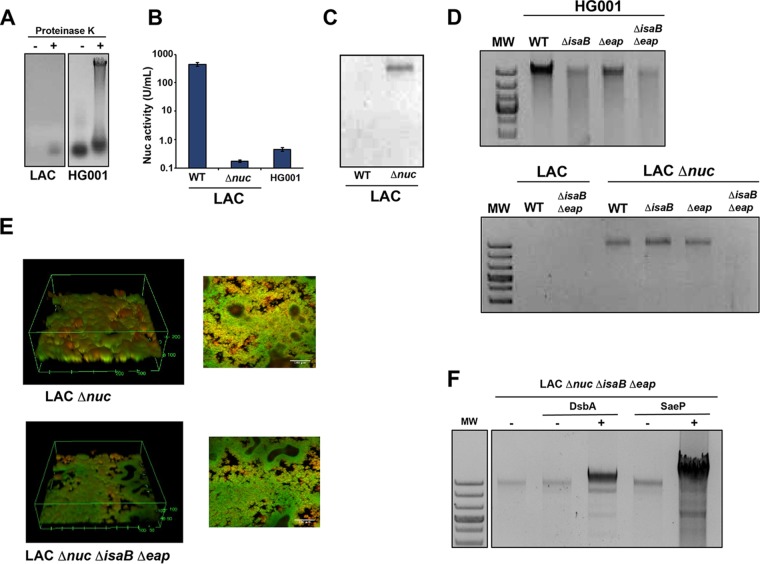
Eap and IsaB impact the surface eDNA and biofilm structure. (A) Release of surface eDNA from HG001 and LAC biofilms with and without proteinase K. (B) Nuc activity, measured by FRET assay, in HG001 and LAC WT and *nuc* mutant strains. (C) Release of surface eDNA from LAC WT and *nuc* mutant biofilms. (D) Release of surface eDNA from *isaB*, *eap*, and double mutants in HG001 and LAC backgrounds. (E) Confocal microscopy images of the LAC *nuc* mutant versus the *nuc isaB eap* triple mutant. (F) Release of surface eDNA from the LAC *nuc isaB eap* triple mutant expressing DsbA or SaeP.

In order for Eap and IsaB functions to be assessed in surface eDNA binding, an assay was developed using approaches pioneered by Kaplan and colleagues (56). We found that proteinase K treatment was critical to the release of the S. aureus eDNA from the surface proteins (Fig. 7A). In our analysis, we focused on high-MW eDNA, since previous work has shown that >11 kb is necessary to function as a biofilm matrix polymer (59). Interestingly, observable amounts of high-MW eDNA could not be recovered from the LAC biofilm matrix, whereas significant amounts of high-molecular-weight DNA were extracted from the HG001 matrix (Fig. 7A), suggesting either that the LAC biofilm does not contain high-molecular-weight DNA or that the DNA is degraded during the extraction process. When the level of Nuc activity was compared between the two strains, LAC produced a high level, typical of USA300 strains (49), while HG001 comparatively produced very little (Fig. 7B), similar to the level of strain SH1000 (50). These observations suggest that Nuc degrades the available high-MW eDNA, and in accordance with this hypothesis, the LAC *nuc* mutant does accumulate high-MW eDNA (Fig. 7C).

With the method described above, we assessed the role of IsaB and Eap in binding surface eDNA using single and double mutant strains. When eDNA was isolated (Fig. 7D), a dramatic reduction in eDNA levels associated with the biofilm matrix was observed when both *eap* and *isaB* were deleted in HG001 or in the LAC *nuc* mutant. A partial loss of eDNA was also observed in the HG001 single mutants. These findings suggest that within the biofilm, Eap and IsaB collectively contribute to the retention of eDNA at the S. aureus surface. Although in the microtiter plate assays the biofilms the LAC strain showed minimal phenotypes (Fig. S4), we reassessed this under flow conditions, where we observed a significant decrease in both overall biomass and the maximum thickness of the LAC *nuc eap isaB* mutant compared to the *nuc* single mutant (Fig. 7E). These results suggest that the loss of eDNA-binding proteins contributes to reduced S. aureus biofilm formation, at least in environments with sheer stress.

Due to the multitude of eDNA-binding proteins identified by the SW blotting method, we moved constructs expressing SaeP and DsbA into the LAC *nuc isaB eap* mutant. A significant increase in matrix-associated eDNA was observed when either of these proteins was expressed at high levels (Fig. 7F), with much greater eDNA retention being observed when SaeP was expressed. The *trans*-complementation approach suggests that secreted eDNA-binding proteins can function in a redundant capacity to retain surface eDNA in the biofilm matrix. Since these strains retain greatly increased levels of eDNA, they would be expected to form biofilms with a greatly reduced porosity. Studies are under way to assess whether this is in fact the case.

### SaeP levels impact nuclease expression.

**FIG 8:**
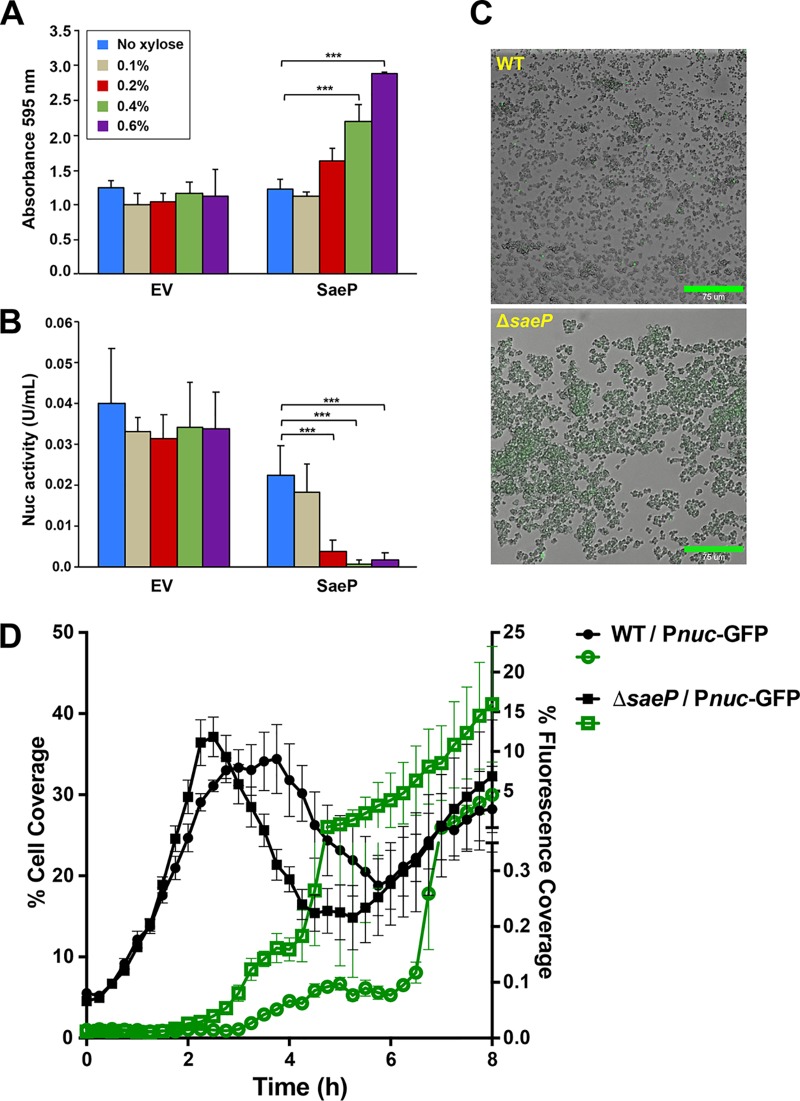
SaeP levels impact Nuc expression. (A) SaeP was overexpressed in LAC under biofilm-forming conditions, and the adherent biomass was quantified using crystal violet. Error bars are the standard deviation for three replicate wells. (B) Biofilm supernatants were filter sterilized and tested for nuclease activity using a Nuc FRET assay. Error bars are the standard deviation for four replicate assays. *P* values were determined by Student’s *t* test. ***, *P* ≤ 0.0005. (C) BioFlux images of the WT and *saeP* mutant with the P*nuc*-GFP reporter. (D) Quantification of the cell coverage and fluorescent coverage of the images in panel C.

Based on studies conducted thus far, it is clear that SaeP is a membrane-anchored lipoprotein that faces the extracellular environment and binds eDNA, thereby enhancing biofilm formation. However, given that SaeP is an auxiliary protein of the SaeRS two-component system that works with SaeQ to modulate the activity of the SaeS sensor kinase (51), it is possible that high levels of SaeP expression could promote biofilm formation by reducing the activity of SaeS and thereby indirectly reducing the levels of secreted Nuc (49, 50). To compare and evaluate these different SaeP functions, Nuc levels were measured during overexpression of SaeP and compared to biofilm growth. As expected, SaeP enhanced the biofilm formation capacity beyond that for the empty vector (Fig. 8A) at xylose levels of 0.2% and above. The same SaeP expression levels corresponded to a decrease in Nuc activity (Fig. 8B), approaching almost nondetectable levels at 0.4 and 0.6% xylose. This observation suggests that SaeP-dependent repression of Nuc activity could impact the ability of S. aureus to form a biofilm; however, the observed levels of Nuc activity within the biofilm (Fig. 8B) were nearly 50-fold lower than the amount of exogenously supplied Nuc that was required to impact biofilm formation (Fig. 5C), suggesting that the regulatory activity of SaeP had at best only a minimal impact in the various static biofilm assays described above. Consistent with this interpretation are the findings that overexpression of SaeP in either a *nuc* mutant (50) or a mutant with a complete deletion of the *saePQRS* operon (46) still resulted in increased biofilm formation (Fig. S3A) and allowed for the extraction of increased levels of high-molecular-weight DNA (Fig. S3B). Collectively, these observations suggest that the enhanced biofilm formation associated with the overexpression of membrane-anchored lipoproteins described above is primarily due to interactions between the lipoproteins and eDNA within the matrix. The observation that the overexpression of SaeP can reduce Nuc activity (Fig. 8B) raises the possibility that at normal levels of expression SaeP can modulate biofilm formation by impacting Nuc expression when Nuc expression is not suppressed by glucose supplementation of the growth medium (49, 50).

10.1128/mBio.01137-19.3 FIG S3 SaeP biofilm enhancement is dependent on SaeRS regulation. (A) The impact of SaeP or DsbA overexpression on biofilm formation was assessed in the LAC wild type, a complete *sae* deletion mutant, and a *nuc* mutant at various xylose concentrations (0% to 0.6%). SaeP overexpression still resulted in significant biofilm enhancement in the *sae* and *nuc* mutants at high levels of induction. (B) The levels of eDNA were assessed in a *nuc* mutant expressing the DsbA control or SaeP (at the indicated xylose inducer levels). *P* values were determined by Student’s *t* test. *, *P* ≤ 0.05; **, *P* ≤ 0.005; ***, *P* ≤ 0.0005. Download FIG S3, JPG file, 1.3 MB. Copyright © 2019 Kavanaugh et al. 2019 Kavanaugh et al. This content is distributed under the terms of the Creative Commons Attribution 4.0 International license.

To examine the impact of SaeP on *nuc* expression, we utilized a BioFlux microfluidics device, which allows for a more comprehensive assessment of biofilm development. Importantly, the growth medium used for this study, 50% tryptic soy broth (TSB), did not contain supplemental glucose, as was the case for the medium used in the various microtiter plate assays described above. Consistent with the above-described experiments indicating a negative effect of SaeP on *nuc* regulation (Fig. 8B), growth of the Δ*saeP* mutant containing the *nuc* reporter plasmid revealed that the stochastic expression observed in the WT strain was nearly eliminated, with the majority of the cells demonstrating *nuc* expression (Fig. 8C and D). In contrast, the temporal control of *nuc* expression appeared to be similar to that of the WT strain, with induction occurring at about 2.5 h after initiation of the experiment. Given these findings, it would appear to be highly plausible that the normal expression levels of SaeP may play a significant role in the regulation of biofilm development. Ongoing studies are aimed at elucidating the function of the SaeRS system and of SaeP in particular in modulating the S. aureus biofilm lifestyle.

### Bioinformatics analysis and perspectives on potential eDNA-binding proteins.

Additional bioinformatics analysis was performed on the many other candidates identified by SW analysis and listed in Table 1 (and Table S1). Literature searches looking for any evidence of DNA-binding activity were conducted, and in addition, the amino acid sequences of hypothetical proteins were submitted for structural prediction on the Phyre2 server (60). As was to be expected, many of the ribosomal proteins identified by the SW analysis are known to interact with nucleic acids, either rRNA, tRNA, or mRNA. Beyond these candidates, bioinformatics structural analysis of two lipoproteins identified in SW analysis band 2, encoded by ORFs 0419 and 1790, suggests that they may bind eDNA with a high affinity.

The 0419 ORF encodes one of nine lipoproteins, referred to as Lpl1 through Lpl9 (also called Csa3A through Csa3I), that are encoded by ORFs 0410 through 0419 on the USA300 chromosome. They are located in the lipoprotein-like (*lpl*) cluster found within the type I vSaα island in S. aureus strains belonging to clonal complex 5 (CC5) and CC8 (61). The nine Lpl proteins share significant sequence homology, with an average percent identity of 61% and a range of identities of 46% to 83%. As a group, the mature Lpl proteins are very basic, with an average theoretical pI of 8.2, suggesting that they should make favorable electrostatic interactions with negatively charged eDNA at neutral pH or under the low-pH conditions found in S. aureus biofilms. While no structures have been determined for the proteins encoded in the *lpl* cluster, sequence homology (Fig. S5A) indicates that they belong to a family of proteins, the conserved staphylococcal antigens (Csa) family (61–63), for which X-ray crystal structures exist (Fig. S5B and C). Importantly, the Csa family includes the protein encoded by ORF 0100, which was shown to bind DNA with a high affinity by EMSA analysis (Fig. 3), which suggests that all of the Csa proteins may bind DNA.

10.1128/mBio.01137-19.5 FIG S5 (A) Alignment of the S. aureus conserved staphylococcal antigens (Csa) family of proteins found in USA300 strain LAC with the Clustal Omega program (89), in which identical residues are highlighted in red and similar residues are shown in red text. The proteins are located within four loci that are indicated at the left of the figure. Locus II is not included in the alignment due to the low sequence homology to the other Csa proteins. The SAUSA300 ORFs are indicated in the first column, and the names of the individual Csa proteins are indicated to the right of the alignment, with the PDB accession numbers being shown in parentheses. (B) Ribbon drawing of Csa4A (PDB accession number 4EGD) showing the concave face of the 10-stranded antiparallel β-sheet with the strand order β6-β7-β8-β9-β10-β1-β2-β3-β4-β5. (C) When the image is rotated 90 degrees, it can be seen that the β-sheet forms a half-barrel that is flanked on its convex side by 4 α-helices. The crystal structures (not shown) for Csa1A (PDB accession number 4BIG [63]) and Csa1B (PDB accession number 4BIH [63]) are nearly identical to the Csa4A structure, and their tertiary structure fold has been designated domain of unknown function 576. (D) The asymmetric unit in the Csa4A crystal structure suggests that the Csa proteins may form split β-barrels in which β-stands 3, 4, and 5 along with α4 form the dimer interface. (E) Electrostatic surface potentials for Lpl proteins encoded by *SAUSA300_0410* and *SAUSA300_0417* calculated over a range of pHs, with positive electrostatic potential shown in blue and negative electrostatic potential shown in red. (F) Electrostatic potentials for all Csa proteins calculated at pH 5. (G) Ribbon drawing of the BR domain of the MSCRAMM protein PsrP from Streptococcus pneumoniae (73) and the electrostatic surface potential calculated at pH 5. Download FIG S5, TIF file, 0.9 MB. Copyright © 2019 Kavanaugh et al. 2019 Kavanaugh et al. This content is distributed under the terms of the Creative Commons Attribution 4.0 International license.

Examination of the available Csa family crystal structures suggests an explanation for the observed high DNA-binding activity of lipoprotein 0100. The RCSB Protein Data Bank (PDB) (64) contains X-ray crystal structures for 0100-encoded Csa1A (PDB accession number 4BIG [63]), Csa1B (PDB accession number 4BIH, [63]), and Csa4A (PDB accession number 4EGD). Affinity pulldown and surface plasmon resonance experiments utilizing purified proteins suggest that Csa paralogs can physically interact with each other (63), and the asymmetric unit in Csa4A (PDB accession number 4EGD) (Fig. S5D) suggests that they may interact to form split β-barrel structures in which β-strands 3, 4, and 5 along with α4 form the dimer interface. Importantly, with an interior diameter in the range of 20 to 25 Å, the barrel is sufficiently large to accommodate a dsDNA helix. Given the high pIs of the Csa proteins noted above and the fact that proteins that utilize β-sheets to bind DNA have positive electrostatic potentials on their surfaces (65), electrostatic surface potentials were calculated for each of the Csa proteins. Atomic models were generated using the Phyre2 server and the structure with PDB accession number 4EGD as a template, and electrostatic potentials were calculated using the PDB2PQR server (66) and PyMOL (The PyMOL molecular graphics system, version 2.1; Schrödinger, LLC). When electrostatic surface potentials were calculated over a range of pHs for two of the Lpls, Lpl1 and Lpl7, the concave surface of the β-sheet was highly positive (Fig. S5E) when the solvent-accessible surface potential was displayed in the same orientation (Fig. S5B). When the electrostatic potentials for each of the Csa proteins were calculated at pH 5 and displayed in the same orientation (Fig. S5F), it is clear that a number of them, including 0100, also had a very positive charge distribution on the concave face of their β-sheets. If the Csa proteins do in fact form split β-barrels, then at the low pH values where S. aureus biofilms form, the interior surface of the barrel would be highly positive, as has been observed for other proteins that use β-sheets to bind DNA (65).

**FIG 9:**
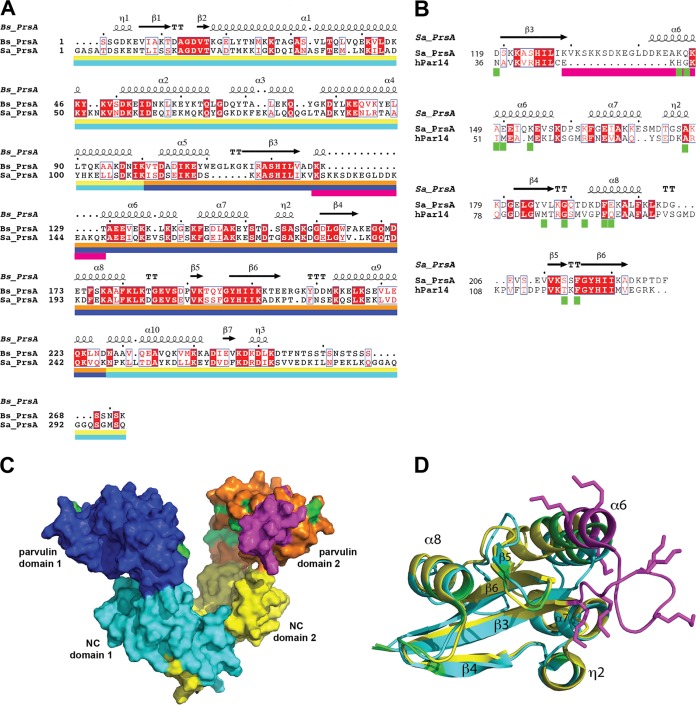
PrsA bioinformatics. (A) Alignment of S. aureus PrsA (Sa_PrsA) with Bacillus subtilis PrsA (Bs_PrsA) determined with the Clustal Omega program (89) and prepared using the ESPript2.2 program (90), in which identical residues are highlighted in red and similar residues are shown in red text. Secondary structure elements from the B. subtilis X-ray crystal structure (PDB accession number 4WO7) are indicated above the sequence, and the color bars below the sequences correspond to the subunit colors in panel C, with the NC and parvulin domains of subunit 1 being indicated by teal and dark blue, respectively, and the NC and parvulin domains of subunit 2 being indicated by yellow and orange, respectively. The magenta bar under parvulin domain 1 indicates the amino acid insertion found in S. aureus PrsA. (B) Alignment of S. aureus PrsA with human parvulin protein hPar14 determined with the Clustal Omega program. The amino acid insertion in S. aureus PrsA is indicated by the magenta bar, and the green bars indicate the hPar14 residues whose NMR chemical shifts were sensitive to the addition of DNA. (C) Image, generated with PyMOL (The PyMOL molecular graphics system, version 2.1; Schrödinger, LLC), showing the molecular surface of the B. subtilis PrsA crystal structure (PDB accession number 4WO7), with NC domain 1 shown in teal, parvulin domain 1 shown in dark blue, NC domain 2 shown in yellow, and parvulin domain 2 shown in orange. The solution NMR structure of the S. aureus parvulin domain (PDB accession number 2JZV) is superimposed on the parvulin domain 2 of B. subtilis, and the extra molecular surface from the amino acid substitution in S. aureus PrsA is shown in magenta. In addition, the molecular surfaces associated with the S. aureus parvulin domain residues corresponding to the human parvulin residues with DNA-sensitive chemical shifts are shown in green. (D) Stereo image depicting the alignment of S. aureus (PDB accession number 2JZV; yellow) and human (PDB accession number 1FJD; teal) parvulin NMR structures, with the amino acid insertion in S. aureus, including the lysine side chains, shown in magenta and the positions of human residues whose chemical shifts are impacted by DNA shown in green.

Structural analysis suggests that the lipoprotein encoded by *SAUSA300_1790* may also promote S. aureus biofilm formation by moonlighting as an eDNA-binding protein. ORF 1790, which encodes the bacterial extracellular foldase PrsA, was identified by SW analysis at a similar high abundance in both membrane band 2 and medium band 10 (Table S1), which is consistent the findings obtained by MS recently reported by Graf et al. (35). PrsA is a highly conserved, essential lipoprotein that contains two subdomains: an NC domain (consisting of the N- and C-terminal portions of the protein) and an intervening parvulin-like domain responsible for the peptide-propyl *cis-trans* isomerase (PPIase) activity of the protein (67). Relative to PrsA in Bacillus subtilis (Fig. 9A) and other Gram-positive bacteria (67), S. aureus PrsA contains a 15-amino-acid insertion in the parvulin domain, which is indicated by the magenta bar in the alignment of the S. aureus and B. subtilis PrsAs shown in Fig. 9A generated with the Clustal Omega program. The parvulin domains of the bacterial PrsAs also share significant sequence homology with human parvulin, as illustrated by the alignment of S. aureus PrsA with human hPar14 (Fig. 9B). Interestingly, S. aureus PrsA contains the same insertion relative to human parvulin as it does relative to other bacterial PrsAs, suggesting that acquisition of the insertion may be a recent evolutionary event. The dimeric X-ray crystal structure of B. subtilis PrsA (PDB accession number 4WO7 [67]) shows a large, bowl-shaped crevice that is roughly 25 Å wide and 40 Å deep, with the NC domains (teal and yellow in Fig. 9C) being the closest to the cell membrane and the parvulin domains (dark blue and orange/magenta in Fig. 9C) being at the top of the bowl. The structure of the complete S. aureus PrsA has not been determined, but a nuclear magnetic resonance (NMR) structure of the parvulin domain (PDB accession number 2JZV) has been published previously (68). In Fig. 9C, the S. aureus NMR structure is superimposed on B. subtilis parvulin domain 2, so that the additional solvent-accessible surface associated with the insertion is shown in magenta on the edge of the bowl, and in Fig. 9D, the S. aureus NMR structure (yellow) is superimposed on the NMR structure (PDB accession number 1FJD [69]) of hPar14 (blue), with the amino acid insertion being shown in magenta. Significantly, hPar14 has been shown to bind short DNA duplexes with dissociation constants (*K_d_*s) of ∼200 nM (70), with the highest affinities (<190 nM) being observed for DNA duplexes containing tracts of 5 to 6 contiguous A residues, which would be common in eDNA derived from low-GC-content S. aureus genomic DNA. By titrating DNA into the hPar14 solution, Surmacz et al. (70) were able to identify residues (indicated by green bars in Fig. 9B) whose chemical shifts were sensitive to the addition of DNA. When surfaces associated with the corresponding residues in the parvulin domain of S. aureus PrsA are colored green (Fig. 9C and D), it can be seen that they are located along the inner face of the crevice/bowl of dimeric bacterial PrsA, suggesting that DNA may bind within the bowl. Interestingly, the insertion that is unique to S. aureus contains several lysine side chains (shown in magenta in Fig. 9D) that would project into the bowl, where they could potentially enhance the ability of S. aureus PrsA to bind DNA relative to other bacterial PsrAs.

## DISCUSSION

In this work, we developed and verified a method for identifying eDNA-binding proteins involved in S. aureus biofilm development. The approach is based on SW blotting methods pioneered to investigate DNA-protein interactions (71). Using the new method, we identified a number of lipoproteins and cell surface-associated proteins that bind DNA, and selected candidates were confirmed and subsequently shown to impact biofilm structure. As anticipated, proteins with known DNA-binding activity were identified, such as the major autolysin Atl, IsaB, Eap, and PSMs, but more interestingly, many uncharacterized proteins were uncovered in the screen. Of these candidates, a number of lipoproteins were detected, and some were investigated further and shown to bind eDNA, positively impact S. aureus biofilm development, and influence biofilm porosity.

The findings reported here complement and extend the electrostatic net model of biofilm development in S. aureus (33, 34). According to this model, cytoplasmic proteins that are released into the extracellular environment through autolysis promote biofilm formation by moonlighting as eDNA-binding proteins. At the acidic pHs associated with biofilm formation, the positively charged proteins can form favorable, attractive electrostatic interactions with both eDNA and the negatively charged teichoic acids found on the cell surface. When the eDNA within the biofilm matrix is of high MW, multiple bacterial cells can associate with any given DNA molecule, promoting cellular aggregation. Since the positively charged proteins also insert between individual DNA molecules, the proteins function as noncovalent links between DNA molecules that would otherwise repulse one another. Therefore, the moonlighting proteins effectively create a network of favorable, attractive electrostatic interactions resembling a net, which promotes cellular aggregation and biofilm development.

Virtually all of the proteins identified by our SW blotting screen (see Table S1 in the supplemental material) were found in the biofilm ECM and/or the biofilm flowthrough in a very recent, comprehensive MS analysis of S. aureus biofilms (35), and roughly 20% of the proteins that we identified were also found in the studies on the electrostatic model (33, 34). By far, the largest differences between the list of proteins that we identified and the proteins identified by these other studies are proteins that we failed to identify as contributing to the biofilm matrix. However, since many of the proteins found to be enriched in biofilms may not have DNA-binding activity, they would have been missed by our approach since we purposely implemented the SW blotting screen prior to MS analysis in order focus on potential eDNA-binding proteins. More significant are the proteins that we identified but that were not identified or that were found at only a low abundance in the other studies. Since we included cell membranes in our study, the SW blotting method allowed for the identification of novel eDNA-binding lipoproteins that are covalently attached to the cell membrane. The others studies relied on salt and increases in pH to release proteins associated with the cell surface through noncovalent interactions, which means that they would be able to find only the lipoproteins that were shed from the cell membrane. At the experimental level, this makes our SW blotting approach complementary to the other studies, but more importantly, it allowed us to uncover the potential roles that these lipoproteins may play in modulating biofilm stability and porosity.

By applying the SW blotting approach to S. aureus membranes, we have extended the electrostatic net model of biofilm development to include membrane-bound lipoproteins that form favorable electrostatic interactions with eDNA and thereby contribute to stabilize the biofilm and modulate biofilm porosity. It seems likely that their contribution to the network has been overlooked due to the methods used, as noted above. Using the SW blotting approach, we identified numerous lipoproteins as potential DNA-binding proteins, and for several of them, we confirmed their DNA-binding activity. EMSA analysis also indicated that the lipoproteins formed very-high-MW complexes that were consistent with nonspecific DNA binding, an important property for proteins that are going to contribute to the electrostatic net by binding eDNA. Running EMSAs at different pHs demonstrated that the lipoprotein DNA-binding activity was driven largely through attractive electrostatic interactions. We further demonstrated that at high levels these lipoproteins were able to enhance biofilm formation. Using SaeP as a test candidate, the enhancement in biofilm capacity was dependent on the presence of eDNA, suggesting that the lipoproteins increase the biofilm formation capacity by making favorable interactions with the eDNA found in the matrix and thereby strengthening the electrostatic net. By quantifying the ability of various-sized FITC-dextrans and proteins to diffuse through biofilms, we provide evidence that these interactions can modulate biofilm porosity. The secreted eDNA-binding proteins identified by the SW blotting screen, for example, Eap and IsaB, likely promote biofilm formation and modulate porosity through the interactions that they make with eDNA in the matrix. All these findings are consistent with the function of the moonlighting proteins that stabilize the biofilm, but our observation that SaeP must be attached to the cell surface provides a new layer to the model. Our observations suggest that lipoproteins may function as anchor points that secure the electrostatic net to the cell surface and that these anchor points are important for promoting biofilm formation and modulating biofilm porosity. The fact that we were able to identify multiple lipoproteins with DNA-binding and biofilm-promoting activity implies a significant level of functional redundancy. Since the bioinformatics analysis suggests that there are likely additional lipoproteins with DNA-binding activity, this redundancy may be even more extensive than that indicated by the results of the SW blotting experiments.

Structural analysis of the candidates identified by the SW blotting screen suggests that additional lipoproteins that were not found during the SW blotting screen may also have DNA-binding activity capable of promoting S. aureus biofilm formation. As noted above, lipoproteins in the Csa family share a structure due to significant sequence homology. Given that Csa lipoprotein 0100 was shown to bind DNA in the EMSA analysis and result in an eDNA-dependent enhancement in biofilm formation when overexpressed (Fig. 3 and 4), it is likely that many of the Csa proteins, including the *lpl* proteins, possess similar DNA-binding activity that could enhance biofilm formation. In this regard, it is interesting to note that it was recently found that lipoproteins having an aspartate at position +2 of the lipobox are more tightly bound to the cell surface (72) and that five of seven lipoproteins with Asp+2 in S. aureus (out of a total of 67 lipoproteins in S. aureus) are part of the *lpl* cluster (see the lipobox sequences in Fig. S5A), suggesting that these proteins could serve as important eDNA-bound anchors. Since analysis of the hypothetical Csa structures suggests that they bind DNA using the positively charged, concave surface of a β-sheet, it is possible that any lipoprotein that presents a positively charged, concave β-sheet on the outside of the cell may form favorable interactions with eDNA. Our findings for lipoprotein 0079 support this notion, as we demonstrated that this protein binds DNA (Fig. 3) and that it results in enhanced biofilm formation when overexpressed (Fig. 4A). Analysis with the Phyre2 server found that 0079 (CopL) has 63% sequence identity (Fig. S6A) to the C-terminal domain of the YdhK protein of unknown function from B. subtilis. The structure of YdhK (PDB accession number 4MDW; Fig. S6B and C) consists of two small β-sheet domains that are connected by two long β-strands (β2 and β5) that form a concave, antiparallel β-sheet that has been designated DUF1541. When the electrostatic surface potential of a 0079 model built on the structure with PDB accession number 4MDW is displayed (Fig. S6B and C), the surface of the cleft between the β-sheet domains is highly positive, suggesting that this surface is likely responsible for the binding DNA observed in the SW and EMSA analyses (Fig. 1 and 3). Of note, it was demonstrated that the binding region (BR) domain of the MSCRAMM protein PsrP from Streptococcus pneumoniae interacts with eDNA to promote bacterial aggregation (73). Interestingly, the BR domain (Fig. S5G) consists of an antiparallel β-sheet that, when dimerized, forms a split β-barrel-like structure in which the positive interior, concave surface of the barrel binds DNA. Collectively, the findings for the Csa proteins, 0079, and PsrP suggest that interactions between eDNA and positively charged, concave β-sheets of lipoproteins may be a general mechanism for stabilizing bacterial biofilms.

10.1128/mBio.01137-19.6 FIG S6 (A) Alignment of the SAUSA300_0079 (CopL) sequence with the sequence of the YdhK protein from Bacillus subtilis, with conserved residues highlighted in red and similar residues shown in red text. (B and C) Ribbon drawings showing the NMR structure of YdhK (PDB accession number 2KY9) in orthogonal views (far left) and with electrostatic surfaces calculated at various pHs for the threaded CopL structure. Regions of positive potential are shown as blue surfaces, and regions of negative potential as shown as red surfaces. Download FIG S6, JPG file, 1.5 MB. Copyright © 2019 Kavanaugh et al. 2019 Kavanaugh et al. This content is distributed under the terms of the Creative Commons Attribution 4.0 International license.

Our discovery of SaeP by the SW blotting approach is of particular significance since this is an auxiliary lipoprotein that works with the membrane protein SaeQ to control the SaeRS two-component system function (51), which raises the possibility that SaeP may have a regulatory function in biofilm formation. While SaeP was predicted to be a lipoprotein, we confirmed that assignment and demonstrated that the C-terminal domain faces the extracellular environment (Fig. 4). This C-terminal domain was found to bind eDNA directly in a pH-dependent manner (Fig. 3), and the expression of SaeP positively impacted the S. aureus biofilm formation capacity. Interestingly, this phenotype was dependent on the availability of eDNA, which, coupled with SaeP localization, allowed us to infer that membrane-bound lipoproteins may function as anchor points between the biofilm matrix and the bacterial cell surface. Through further investigation, the positive impact of SaeP expression on biofilm formation was also linked to its function as a repressor of the SaeRS system (50). The repression of Nuc activity as a consequence of SaeP overexpression correlated with increased amounts of higher-molecular-weight eDNA being associated with the cell surface and biofilm matrix. Consistent with a regulatory role for SaeP, we found that inactivation of *saeP* resulted in elevated *nuc* expression levels (Fig. 8D) and relieved the bistability phenotype that we previously demonstrated for *nuc* expression using the BioFlux device (19). This observation demonstrates that SaeP modulates SaeRS function and controls the stochastic response normally observed in wild-type strains. Taken together, this raises the possibility that SaeP may regulate biofilm formation and porosity by modulating the local availability of high-molecular-weight eDNA.

In this work, we developed a novel SW blotting screen for the discovery of S. aureus biofilm matrix proteins with DNA-binding activity. Collectively, our findings make a case for extending the electrostatic net model for S. aureus biofilm formation to include lipoproteins. These membrane-attached lipoproteins may function as important anchor points that connect the bacterial cell surface to the biofilm matrix. Although our screen was limited to S. aureus, our findings should be applicable to many different bacteria since eDNA is recognized as a structural component of the biofilm matrix for numerous bacterial species (74–76). Moreover, given that biofilms are implicated in various types of invasive and chronic infections, our findings suggest that lipoproteins make attractive targets for novel antibiofilm therapies aimed at disrupting biofilms by interfering with the interactions between individual bacteria and the biofilm matrix.

## MATERIALS AND METHODS

### Bacterial strains and growth conditions.

Escherichia coli was grown in Luria-Bertani (LB) and S. aureus was grown in tryptic soy broth (TSB) at 37°C with shaking unless otherwise stated. For a complete list of strains, see Table 2. The antibiotic concentration for maintenance of E. coli plasmids was 100 μg ml^−1^ for ampicillin (Amp) and 50 μg ml^−1^ for kanamycin (Kan). S. aureus plasmids were maintained with 10 μg ml^−1^ chloramphenicol (Cam) or 10 μg ml^−1^ erythromycin (Erm). Bacterial growth was monitored using a Tecan Infinite M200 plate reader (Tecan, Switzerland).

**TABLE 2:** Strains and plasmids used in this study

Strain	Genotype	Reference or source
AH1263	USA300 CA-MRSA strain LAC Erm^s^ isolate	79
AH1178	Newman	91
AH2900	Newman Δ*eap*	42
AH2902	Newman Δ*eap*/pLL39_*eap*	42
AH1811	LAC (AH1263) *hla*::*erm*	43
AH2216	LAC (AH1263) Δ*saePQRS*	46
CFS22	AH2216/pEPSA5_*saeP*	This study
CFS159	AH2216/pEPSA5	This study
AH2340	E. coli ER2566/pET28a_*saeP*	This study
AH3856	E. coli ER2566/pET28a_0079	This study
AH3858	E. coli ER2566/pET28a_0100	This study
AH3860	E. coli ER2566/pET28a_2354 (*dsbA*)	This study
AH3498	LAC (AH1263) Δ*saeP*	This study
CFS155	LAC (AH1263)/pEPSA5_*saeP*-T7	This study
CFS227	LAC (AH1263)/pEPSA5_0079-T7	This study
CFS228	LAC (AH1263)/pEPSA5_0100-T7	This study
CFS	LAC (AH1263)/pEPSA5_2354 (*dsbA*)-T7	This study
CFS229	LAC (AH1263)/pEPSA5_0175-T7	This study
CFS230	LAC (AH1263)/pEPSA5_1436-T7	This study
CFS231	LAC (AH1263)/pEPSA5_1478-T7	This study
CFS47	AH2216/pEPSA5_*saeP*(C133A)-T7	This study
CFS222	AH2216/pEPSA5_*saeP*(C21A)-T7	This study
CFS210	LAC(AH1263) *atl*::Tn/pEPSA5_*saeP*-T7	This study
AH1570	LAC(AH1263)/pCM20	
AH3759	LAC(AH1263)/pCM38 (Pnuc_sGFP)	This study
AH3760	LAC(AH1263) Δ*saeP* pCM38 (Pnuc_sGFP)	This study
AH4333	LAC(AH1263) *nuc*::LtrB/pEPSA5_*saeP*	This study
AH4334	LAC(AH1263) *nuc*::LtrB/pEPSA5_*dsbA*	This study
AH2582	LAC(AH1263) Δ*isaB*	This study
AH3201	LAC(AH1263) *eap*::*erm*	This study
AH3202	LAC(AH1263) Δ*isaB eap*::*erm*	This study
AH2183	HG001	53
AH2500	AH2183 Δ*isaB*	This study
AH3513	AH2183 *eap*::erm	This study
AH3514	AH2183 Δ*isaB eap*::*erm*	This study
AH3611	LAC(AH1263) Δ*isaB eap*::*erm nuc*::LtrB	This study
AH4791	LAC(AH1263) Δ*lpl* (Δ*SAUSA300_0400-0409)*	This study
AH5275	LAC(AH1263) Δ*csa locus I* (Δ*SAUSA300_0100-0103)*	This study
AH5394	LAC(AH1263) Δ*csa locus I* Δ*lpl* Δ*csa locus IV*	This study
AH5409	LAC(AH1263) Δ*copL*::Tn (Δ*SAUSA300_0079*)	This study

### Recombinant DNA and genetic techniques.

Restriction enzymes were obtained from New England BioLabs (Beverly, MA), and T4 DNA ligase was obtained from Promega Corporation (Madison, WI). The primers used in this study are listed in Table S2 in the supplemental material and were synthesized at Integrated DNA Technologies (IDT; Coralville, IA). DNA manipulations were performed in E. coli strain ER2566 or GM2163 (New England BioLabs). Plasmids from ER2566 were transformed into S. aureus strain RN4220 by electroporation as previously described (77). Plasmids from GM2163 were electroporated directly into strain LAC-derived strains as described previously (78). Chromosomal markers were moved by bacteriophage ϕ11 transduction, and strain constructions were verified by PCR. S. aureus genomic DNA was isolated using a Puregene DNA purification kit (Qiagen). Plasmid constructs and S. aureus mutants were confirmed by DNA sequencing at the University of Iowa DNA Core Facility or at the University of Colorado Barbara Davis Center for Diabetes.

10.1128/mBio.01137-19.8 TABLE S2 Primers and plasmids used in this study. Download Table S2, DOCX file, 0.02 MB. Copyright © 2019 Kavanaugh et al. 2019 Kavanaugh et al. This content is distributed under the terms of the Creative Commons Attribution 4.0 International license.

### Growth curves.

Overnight cultures of strains were diluted 1:100 into 25 ml tryptic soy broth (TSB) in 125-ml flasks and incubated in a 37°C shaker at 225 rpm. At each time point, 200 μl was removed and placed in a 96-well plate. The optical density at 600 nm (OD_600_) was measured in a Tecan Infinite M200 plate reader (Tecan, Switzerland).

### Construction of strains. (i) *ΔsaeP* mutant.

Two-step overlap PCR was used to create pJB38-Δ*saeP*. Regions of 600 bp directly upstream and downstream of *saeP* were amplified from AH1263 genomic DNA (79) with primer pairs MJT240/CEF169 and CEF170/CEF171 respectively, where CEF169 and CEF170 contained complementary overlapping regions (Table S2). The PCR products were purified, mixed, and used as the template for the second round of PCR with primer pair MJT240/CEF171. The 1.2-kb PCR product was purified, digested with EcoRI and KpnI, and ligated into similarly digested pJB38 (80). The resulting plasmid was used to construct a markerless Δ*saeP* strain in the AH1263 background, as described above, and the strain was named CEF93.

**(ii) *ΔisaB* mutant.** As described above, a two-step overlap PCR was used to create pJB38-Δ*isaB*. Regions directly upstream and downstream of *isaB* were amplified from AH1263 genomic DNA with primer pairs CBR2/5 and CBR3/6, respectively (Table S2). The PCR products were purified, mixed, and used as the template for the second round of PCR with primer pair CBR5/6. The final PCR product was purified, digested with EcoRI and KpnI, and ligated into similarly digested pJB38. The resulting plasmid was used to construct a markerless Δ*isaB* strain in the AH1263 background, and the strain was named AH2582.

**(iii) Csa deletion mutants.** Markerless mutations of genes or gene clusters were introduced using the temperature-sensitive plasmid pJB38 carrying DNA fragments (∼1 kb in size) flanking the region targeted for deletion. The flanking regions were amplified by Phusion High-Fidelity DNA polymerase (New England BioLabs) with the primers listed in Table S2. The PCR products and pJB38 were digested with restriction enzymes, as indicated in Table S2, and subsequently purified with a QIAquick PCR purification kit (Qiagen, Germany). For the flanking region upstream of the *SAUSA300_0410* gene, an EcoRI site located 51 nucleotides downstream of the start of the cloned flanking region was used for restriction. After triple ligation of the flanking region pairs with pJB38, the resulting plasmid was electroporated into E. coli DC10B (78). E. coli cells carrying the plasmid were selected on LB plates containing 100 μg/ml ampicillin, single colonies were picked, and the presence of the plasmid was confirmed by PCR using the primers listed in the Table S2. The plasmid was recovered from overnight cultures of positive clones with a QIAquick Spin miniprep kit (Qiagen, Germany), and the sequence of the flanking regions was confirmed by in-house sequencing with their respective construction primers and sequencing primers listed in Table S2. The plasmid was electroporated into the S. aureus target strain, positive clones carrying pJB38 with the desired flanking regions were selected on tryptic soy agar (TSA) plates containing 10 μg/ml chloramphenicol (Cam) at 30°C, and the presence of the plasmid was confirmed by PCR with the primers listed in Table S2. For homologous recombination, positive clones were streaked on TSA-Cam and incubated at 42°C for 24 h. Cam-resistant colonies were restreaked on TSA-Cam and incubated at 42°C overnight. Single colonies were picked and incubated in 5 ml TSB at 30°C and 200 rpm overnight. The resulting overnight cultures were diluted 1:1,000 in TSB and incubated at 30°C and 200 rpm overnight. This was repeated for six consecutive days, and subsequently, dilutions (10^−6^, 10^−7^, and 10^−8^) were plated on TSA plates containing 200 ng/ml anhydrotetracycline for counterselection and incubated at 30°C overnight. Single colonies were then patched on TSA and TSA-Cam plates and grown at 30°C overnight. Plasmid loss was indicated by growth on TSA but not TSA-Cam. Colonies were screened for the desired mutation by PCR with primers located on the S. aureus chromosome outside of the mutated region (Table S2). Mutation of locus I was achieved by deletion of the genes *SAUSA300_0100* to *SAUSA300_0103*, mutation of *lpl* was achieved by deletion of the genes *SAUSA300_0410* to *SAUSA300_0419*, and mutation of locus IV was achieved by consecutive deletion of the genes *SAUSA300_2429*, *SAUSA300_2430*, and *SAUSA300_2424*.

**(iv) CopL mutant.** The transposon mutation for CopL (mutant NE1913) was moved into strain LAC from the Nebraska Transposon Mutant Library (81) by phage transduction using ϕ11.

### Plasmid construction. (i) pSKerm-saePQRS complementation plasmid and C133A.

The full *sae* operon (*saePQRS*), including 300 bp upstream of *saeP*, was amplified from AH1263 genomic DNA using primers CEF16 and CEF17. The 3.5-kb PCR product was purified by agarose gel electrophoresis, digested with HindIII and XmaI, and ligated to pSKerm, which had been digested with the same enzymes (49). The SaeP C133A point mutation was generated using two-step overlap PCR with vector primers (pSKerm for/pSKerm rev) and complementary mutagenesis primers (CEF27/CEF28). All constructs were confirmed by sequencing at the University of Iowa DNA Core Facility.

**(ii) pEPSA5_SaeP-T7 and point mutants.** To construct a protein expression construct of SaeP with the superoxide dismutase ribosome-binding site and a C-terminal T7 tag, *saeP* was amplified from AH1263 genomic DNA using primer pair CEF64/CEF65. The PCR product was digested with EcoRI and XhoI and ligated to similarly digested pEPSA5 (45). The C21A point mutation was generated using two-step overlap PCR with vector primers and complementary mutagenesis primers. The 5′ fragments were amplified using pEPSA5_*saeP* as a template and an upstream vector primer (pEPSA5for2) and reverse primer containing the alanine substitution (CEF227). The 3′ *saeP*-T7 fragments were amplified using the same template with forward primers containing alanine substitutions (CEF226) and a downstream vector primer (pEPSA5rev). The PCR products were purified, and appropriate upstream and downstream fragments were mixed in a 1:1 ratio. The mixtures were used as templates for the second round of PCR with primer pair EPSA5for2/EPSA5rev. Overlap PCR products were purified, digested with EcoRI and XhoI, and ligated to pEPSA5 that had been digested with the same enzymes. All mutants were confirmed by sequencing at the University of Iowa DNA Core Facility.

**(iii) pEPSA5_DsbA-T7.** A C-terminally T7-tagged lipoprotein control was constructed by amplifying *dsbA* from AH1263 genomic DNA using primer pair CEF217/CEF218. The PCR product was digested with EcoRI and XhoI and ligated to a similarly digested pEPSA5.

### Southwestern analysis.

A screen based on SW blotting techniques typically used for characterizing protein-DNA interactions was developed to identify proteins capable of interacting with eDNA (71). To generate protein samples for analysis, biofilm and planktonic cultures of either the wild type or a deletion mutant were grown overnight. Biofilms were grown in plasma-coated plates as previously described (82), and planktonic cultures were grown in TSB. Biomass was collected by pelleting planktonic cells or scraping off biofilm cells, and spent medium was filter sterilized and stored at −20°C until the SW analysis was performed. Isolation of membrane-associated proteins was conducted as previously described (83). Briefly, cells were washed twice with 1× SMM buffer (0.5 M sucrose, 20 mM maleic acid, pH 6.5, 20 mM MgCl_2_) and then resuspended in SMM buffer containing 10 μg/ml of lysostaphin (AMBI Products, Lawrence, NY) and incubated for 1.5 h at 37°C. Protoplasts were harvested by centrifugation (12,000 × *g* for 15 min at 4°C), washed with SMM buffer, and resuspended in 20 mM Tris, pH 8.0. Protoplasts were lysed by sonication on ice (3 times for 5 min each time, 50% duty) using a Sonifier 450 apparatus (Branson, Danbury, CT). Following centrifugation to remove insoluble cell debris (15,000 × *g* for 20 min at 4°C), the membranes were pelleted by centrifugation at 100,000 × *g* for 90 min at 4°C. The membrane pellets were washed twice with 20 mM Tris, pH 8.0, and resuspended in the same buffer. The protein concentrations of the samples were determined by the Bradford assay, and equal amounts of protein were separated by SDS-PAGE using duplicate 4 to 20% nonreducing gradient gels (Bio-Rad Laboratories, Hercules, CA, USA). One gel was stained with Coomassie for protein band visualization, and the second gel was incubated in refolding TNDCaMg buffer (10 mM Tris, pH 7.5, 50 mM NaCl, 1 mM dithiothreitol, 5 mM CaCl_2_, 5 mM MgCl_2_) containing 0.9 mM Triton X-100 for two 6-h washes to allow for protein refolding within the gel. The gel was then incubated for 2 h at room temperature in TNDCaMg buffer (without Triton X-100) containing 2.5 nM an IRD700-labeled 259-bp dsDNA probe. The probe was generated from the *nanA* promoter region (84) using IRD700-labeled primers (IDT, Coralville, IA) MO122IRD700 and MO123IRD700 and purified by electroelution. Following incubation, both gels (the Coomassie-stained and IRD700-DNA-probed gels) were imaged using the 700-nm channel of a LI-COR Odyssey CLx imager (LI-COR, Omaha, NE) and processed using Image Studio software. The Coomassie-stained and DNA-probed gel images were aligned to identify protein bands with DNA-binding activity. These bands were excised and analyzed at the University of Iowa Proteomic Facility. Following elution from the gel, the proteins were digested with trypsin, and the resulting peptides were centrifuged through a 3-kDa-molecular-wright-cutoff membrane and lyophilized. The tryptic peptides were reconstituted and separated with an acetonitrile-water gradient using reversed-phase high-performance liquid chromatography on a Nanobore C_18_ column coupled on-line to a high-resolution tandem mass spectrometer (MS-MS). Mass spectrometric analyses were conducted on a quadrupole time of flight (Q/TOF) mass spectrometer (Agilent 6520) with a nanospray ionization source. The mass spectral data were searched against a library of staphylococcal tryptic peptides using Scaffold (version 4.8.4) proteome software (Proteome Software, Inc., Portland, OR).

### Electromobility shift assays.

A portion of each purified lipoprotein was dialyzed into either Tris-acetate, ethanolamine-CAPSO (3-(cyclohexylamino)-2-hydroxy-1-propanesulfonic acid), or bis-Tris–ACES (*N*-(2-acetamido)-2-aminoethanesulfonic acid) pK_a_-matched buffer systems with NaCl added to 155 mM so that they could be used in electromobility shift assays at pHs 8.0, 9.6, and 6.7, respectively. For each buffer system, a serial 2-fold dilution series of each lipoprotein (20 to 2.5 μM) was combined with an IRD700-labeled DNA probe (the same 259-bp probe used for SW analysis) at a final concentration of 5 nM, incubated for 30 min at room temperature, and then electrophoresed on 1% agarose gels that had been cast with the appropriate pK_a_-matched buffer. Following electrophoresis, gels were scanned using the 700-nm channel on an Odyssey CLx imager (LI-COR, Omaha, NE) and visualized using Image Studio software.

### SCAM.

The substituted cysteine accessibility method (SCAM) was performed with S. aureus strain AH2216 according to previously published methods with minor modifications (52). In brief, overnight cultures were diluted 1:50 into TSB containing Cam and 0.1% xylose and grown in a 37°C shaker until the cultures reached an OD_600_ of 2.0. The cells were pelleted and resuspended to an OD_600_ of 25 in buffer A (100 mM HEPES, 250 mM sucrose, 25 mM MgCl_2_, 0.1 mM KCl, pH 7.5). One set of samples was pretreated with lysostaphin (AMBI Products) prior to labeling with *N*^•^-(3-maleimidylpropionyl)biocytin (MPB; Invitrogen). The other set of samples were first treated with MPB, followed by incubation with lysostaphin. Residues exposed to the extracellular milieu label under all conditions, while residues in the cytoplasm are accessible only following permeabilization of the cells with lysostaphin. Both sets of samples were solubilized by adding 400 μl T7 binding/wash buffer (4.29 mM Na_2_HPO_4_, 1.47 mM KH_2_PO_4_, 2.7 mM KCl, 137 mM NaCl, 1% NP-40 substitute, pH 7.3). Labeled SaeP constructs were immunoprecipitated using anti-T7 agarose (Novagen) and eluted with T7 elution buffer (100 mM citric acid, pH 2.2, 0.5% SDS). To avoid possible aggregation, samples were not boiled following addition of 2× SDS-PAGE loading buffer containing 5% β-mercaptoethanol before analysis by anti-T7 and Strep-HRP Western blotting.

### Forty-eight-well microtiter biofilm assays.

Overnight cultures were diluted 1:500 in TSB containing 0.4% glucose and appropriate antibiotics. Subcultured bacteria were incubated in a 37°C shaker for 30 min prior to inoculating 1 ml per well in a 48-well plate. Xylose was added to the appropriate wells at the specified concentrations, and the plates were incubated in a 37°C humidified shaker (Stuart SI505; Techni Inc., Burlington, NJ) at 500 rpm for 24 h, unless specified otherwise. Biofilms were stained by first aspirating the supernatant from each well using a 26-gauge needle. Nonadherent cells were removed by washing with 750 μl distilled H_2_O (dH_2_O) with a multichannel pipette. To stain the adherent biomass, 500 μl of 0.1% crystal violet was added to each well and the plate was incubated at room temperature for 10 min. Crystal violet was removed by aspiration, and the biofilms were washed with 750 μl dH_2_O with a multichannel pipette. To solubilize the remaining crystal violet, 1 ml isopropanol was added to each well and the plate was incubated at room temperature for at least 30 min. One hundred microliters of solubilized crystal violet was mixed with 100 μl of isopropanol in a 96-well plate, and the plate was read at OD_595_ in a Tecan Infinite M200 plate reader.

### Ninety-six-well microtiter biofilm assays.

An overnight culture of AH1263 was diluted 1:500 in TSB containing 0.4% glucose. Subcultured bacteria were incubated in a 37°C shaker for 30 min prior to inoculating 200 μl to a 96-well plate. Purified His-SaeP (described below) was added to each well at the prescribed amount, and the plates were incubated in a 37°C humidified shaker at 500 rpm for 24 h. Biofilms were stained by first aspirating the supernatant from each well using a 26-gauge needle. Nonadherent cells were removed by washing with 200 μl dH_2_O with a multichannel pipette. To stain the adherent biomass, 100 μl of 0.1% crystal violet was added to each well and the plate was incubated at room temperature for 10 min. Crystal violet was removed by aspiration, and the biofilms were washed with 200 μl dH_2_O with a multichannel pipette. To solubilize the remaining crystal violet, 200 μl isopropanol was added to each well and the plate was incubated at room temperature for at least 30 min. One hundred fifty microliters of solubilized crystal violet was transferred to a new 96-well plate, and the plate was read at OD_595_ in a Tecan Infinite M200 or Tecan Infinite 200Pro plate reader.

### Biofilm porosity measurements.

Biofilms were grown in grown in 96-well filter plates that used PVDF membranes with 0.45-μm pores (Agilent). Overnight cultures, grown in TSB, were inoculated at a ratio of 1:250 into TSB supplemented with 0.6% yeast extract and 0.8% glucose, and 100-μl aliquots were dispensed into plate wells that had been prefilled with 100-μl aliquots of TSB supplemented with 0.6% yeast extract and 0.8% glucose and that contained either no methicillin or methicillin at 4 μg/ml, yielding a final concentration of 2 μg/ml for wells containing methicillin. Identical biofilms were set up in parallel using standard 96-well microtiter plates. Wells containing only sterile medium were included on the filter plates in order to provide no-biofilm control wells for porosity measurements. After the plates were incubated statically for 24 h at 37°C, the standard plates were stained with crystal violet as described above and the filter plates were used for measuring biofilm porosity. Culture supernatants were removed by aspiration and 100 μl of buffer (50 mM MES [morpholineethanesulfonic acid], pH 5.5, 150 mM NaCl) containing either an FITC-isocyanide-dextran (with dextran at a molecular weight of either 4K, 10K, 70K, or 150K) at 1 mg/ml or 100 μl of MES buffer containing a mixture of bovine serum albumin (BSA; MW, 66 kDa), yeast alcohol dehydrogenase (ANH; MW, 150 kDa), and sweet potato β-amylase (MW, 200 kDa) at concentrations of 1, 12.5, and 12.5 mg/ml, respectively. The higher concentrations of ADH and β-amylase were used in order to ensure that the proteins were fully in their tetrameric states. The filter plates were centrifuged in a swinging-bucket rotor for 45 s at 20 × *g*, which resulted in ∼5 to 10 μl of flowthrough in the biofilm plates and ∼75 μl of flowthrough in the wells with sterile medium. The concentrations of FITC-dextran in the biofilm flowthrough fractions were quantified by transferring 5 μl of flowthrough to 96-well microtiter plates in which the wells had been prefilled with 45 μl of MES buffer (that did not contain FITC-dextran), which resulted in a sufficient volume to uniformly cover the bottoms of the wells and a FITC concentration that was still within a linear concentration, as confirmed by standard curves, when the fluorescence was measured in a Tecan Infinite 200Pro plate reader using a gain of 100 and excitation and emission wavelengths of 470 nm and 523 nm, respectively. The relative concentrations of proteins in the biofilm flowthrough fractions were determined by separating the proteins by SDS-PAGE using 4 to 20% gradient gels (Bio-Rad) and staining with Bio-Safe Coomassie G250 stain (Bio-Rad), followed by integration of band intensities by scanning of the gels at 700 nm using a LI-COR Odyssey CLx scanner and Image Studio software. Analysis of the flowthrough data was accomplished using Excel and Prism software. The approaches of quantifying the flowthrough of both FITC-dextrans and proteins have distinct and complementary advantages and disadvantages. FITC-dextrans have the disadvantage that only a single-molecular-weight dextran can be passed through each well, but the simplicity of the method lends itself to examining large numbers of wells. In contrast, the use of proteins has the disadvantages of being more cumbersome and having intrinsically higher error rates due to the increased number of steps in the process; it has the advantage of being able to pass proteins with multiple molecular weights through the same biofilm. We therefore chose to use both methodologies to assess biofilm porosity.

### Flow cell biofilms.

Flow cell biofilms were conducted as previously reported with minor modifications (50). Briefly, overnight cultures were diluted 1:100 in TSB. Inverted flow cell chambers were inoculated with 1 ml of the subcultures. Cells were allowed to adhere to the acid-etched glass coverslips for 1 h at room temperature. Following attachment, flow was initiated at a rate of 3.5 rpm. The biofilm medium consisted of 2% TSB supplemented with 0.2% glucose and 1 μg/ml of chloramphenicol. For strains containing pEPSA5 as either the empty vector or the vector expressing lipoprotein, the medium was supplemented with 1 μg/ml of chloramphenicol and 0.1% xylose. The biofilms were grown at 37°C for 2.5 days. Live/dead staining was performed on mature biofilms using SYTO9 and propidium iodine at concentrations of 2.5 μM and 15 μM, respectively (LIVE/DEAD *Bac*Light bacterial viability kit; Invitrogen). Biofilms were imaged using a Leica scanning confocal laser microscope, and three representative images were taken for each chamber. Images were processed using the FIJI platform (85), and statistical analysis was conducted using COMSTAT2 software (86, 87).

### Western blotting.

Samples were electrophoresed on 15% polyacrylamide gels in a Protean 3 system (Bio-Rad Laboratories, Hercules, CA, USA). Proteins were transferred to Immobilon-P PVDF membranes (Millipore) at 170 mA for 1 h. For detection of T7-tagged SaeP proteins, membranes were blocked overnight at 4°C with 5% milk in Tris-buffered saline (TBS; 20 mM Tris-HCl, pH 7.0, with 137 mM NaCl) containing 0.05% Tween 20 (TBST). Anti-T7 antibody conjugated to horseradish peroxidase (HRP; Novagen) was diluted 1:15,000 in 0.05% TBST and incubated with the membranes at room temperature for 1 h. The membranes were washed five times for 2 min each time with 0.05% TBST. For the detection of biotinylated proteins from the SCAM assays, the membranes were blocked overnight at 4°C with 3% bovine serum albumin in 0.05% TBST. After three 10-min washes, the membranes were incubated with streptavidin-HRP (1 mg/ml; Thermo Scientific) diluted 1:7,500 in 0.05% TBST at room temperature for 1 h. The membranes were washed three times for 10 min each time with 0.05% TBST. All membranes were imaged by incubating with the SuperSignal West Pico chemiluminescent substrate (Thermo Scientific) for 1 min and then exposed to X-ray film (Research Products International Corporation).

### Cloning and purification of His-tagged lipoproteins.

Soluble versions of lipoproteins SaeP (SAUSA300_0693), SAUSA300_0079, SAUSA300_0100, and DsbA (SAUSA300_2354) lacking the N-terminal secretion signal and the Cys residue that would be modified with lipid were overexpressed in E. coli and purified by Ni-affinity chromatography. The genes were amplified from strain LAC genomic DNA using Phusion high-fidelity DNA polymerase (New England Biolabs, Ipswich, MA) and the following pairs of primers: for SaeP, primers CEF43 and CEF44; for SAUAS300_0079, primers 00795 and 00793; for SAUSA300_0100, primers 01005 and 01003; and for DsbA, primers 23545 and 23543. The PCR products were digested with NheI-HF and BamHI-HF and ligated using T4 DNA ligase into similarly digested pET28a that had been treated with shrimp alkaline phosphatase. E. coli expression strain ER2566 was transformed with the ligations, and positive transformants were confirmed by DNA sequencing and tested for protein expression. For purification, 2 liters of LB medium containing kanamycin at 50 μg/ml was inoculated with 5 ml of an overnight culture of the lipoprotein-expressing E. coli strain and grown at 37°C with shaking at 250 rpm to an OD_600_ of 0.5 to 0.6. Expression was induced with 1 mM IPTG (isopropyl-β-d-thiogalactopyranoside), and the flasks were incubated at 30°C for 4 h. Cells were harvested by centrifugation and resuspended in His-Select resin (Sigma) equilibration buffer (50 mM sodium phosphate, 300 mM NaCl, 10 mM imidazole, pH 8). Cells were lysed using BugBuster master mix (Novagen), and the resulting lysates were cleared by centrifugation at 30,000 × g for 30 min at 4°C before loading onto columns containing His-Select resin (Sigma). The columns were washed with at least 10 column volumes of equilibration buffer, and proteins were eluted with 2 column volumes of elution buffer (50 mM sodium phosphate, 300 mM NaCl, 250 mM imidazole, pH 8). The purified proteins were dialyzed into phosphate-buffered saline (PBS).

### Nuc FRET assays on biofilm supernatant.

The Nuc Förster resonance energy transfer (FRET) assay was performed on biofilm supernatants as previously described with minor modifications (50). The FRET oligonucleotide was diluted to 4 μM in 20 μM Tris, pH 8.0, 10 μM CaCl_2_. Twenty-five microliters of FRET substrate was mixed with 25 μl of undiluted biofilm supernatant in a 96-well plate using a multichannel pipette. Solutions were mixed vigorously while avoiding air bubbles and then immediately read in a Tecan Infinite M200 plate reader at 552 nm of excitation, 580 nm of emission, and a gain of 100 every 3 s for several minutes. Fluorescence units were plotted versus time, and the plot was fit with a linear fit to determine the slope, or the rate of the reaction. The FRET assay was repeated four times, and the number of units per milliliter was calculated.

### Nuc prevention/dispersal of SaeP biofilms.

Purified nuclease (Worthington Biochemical Corporation, Lakewood, NJ) was resuspended in double-distilled H_2_O (ddH_2_O) at a concentration of 400 U/ml. Fourfold serial dilutions were performed in ddH_2_O, and 20 μl of each dilution was added across a 96-well plate. One concentration of xylose was added across each row containing a dilution series of nuclease, such that each row had either 0, 0.2%, 0.4%, or 0.6% xylose. Overnight cultures of AH2816 (AH1263/pEPSA5) and CFS155 (AH1263/pEPSA5_SaeP-T7) were diluted 1:500 in TSB containing 0.4% glucose. Subcultured bacteria were incubated in a 37°C shaker for 30 min, prior to inoculating 180 μl into each well of the prepared 96-well plate containing various concentrations of nuclease and xylose. The plates were incubated in a 37°C shaker at 225 rpm for 24 h. The biofilms were stained as described above (see “Ninety-six-well microtiter biofilm assays” above).

### eDNA isolation assay.

Biofilm matrix-associated eDNA was isolated as previously described with some modifications (56). Overnight cultures were diluted 1:1,000 in TSB supplemented with 0.4% glucose. Six-well plates were inoculated with 4 ml per well of subcultures, and appropriate amounts of xylose were added to the wells. The biofilms were grown statically for 16 h in a humidified 37°C shaker. To collect the biofilms, the supernatant was first aspirated off using a 26-gauge needle. The biofilms were then removed using a cell scraper and placed into preweighed microcentrifuge tubes, and the biomass was recorded. The biofilms were resuspended in PBS to 100 mg of biomass/ml. Proteinase K was added at 5 μg/ml, and samples were incubated at 37°C for 2 h to facilitate eDNA release, as previously reported (88). Cells were removed through centrifugation, and equal volumes of supernatant were collected. eDNA was isolated using ethanol precipitation (2.5 volumes of 100% ethanol, 1/10 volume of 3 M sodium acetate, pH 5.0). eDNA was pelleted by centrifugation and resuspended in 200 μl TE (Tris-EDTA) buffer. Samples were analyzed using agarose gel electrophoresis and ethidium bromide staining.

### BioFlux1000 biofilm assays.

A BioFlux1000 microfluidic system (Fluxion Biosciences, Inc., San Francisco, CA) was used to assess biofilm development as previously described (19). BioFlux1000 48-well plates were used for all experiments. Biofilm growth channels were primed by adding 200 μl of 50% TSB to the output wells and using a reverse flow for 5 min at 5.0 dynes/cm^2^. The TSB in the output wells was replaced with 300 μl of fresh inoculum made from overnight-grown S. aureus cultures diluted to an optical density at 600 nm (OD_600_) of 0.8. Three hundred microliters of fresh TSB was added to the input wells. The growth channels were then seeded by applying a reverse flow for 2 s at 2.0 dynes/cm^2^. The seeded plate was left to incubate on the heated (37°C) stage of the BioFlux1000 system for 1 h to allow cells to attach to the growing channel walls. Inoculum remaining in the input and the output wells was aspirated, and 1.3 ml of fresh 50% TSB (with or without 2% xylose for the *saeP*-overexpressing strain) was added to the input wells. A forward flow at 0.6 dyne/cm^2^ was then applied to the channels for 18 h. Bright-field and epifluorescence images were taken in 5-min intervals for a total of 217 time points. All epifluorescence images monitoring green fluorescent protein (GFP) expression were acquired using a fluorescein isothiocyanate (FITC) filter. Acquisition settings for images were kept constant throughout all experiments (bright-field exposure, 10 ms; FITC exposure, 10 ms).

### Quantification of acquired biofilm assay images.

Images representative of the phenotypes recorded were observed and selected using BioFlux Montage software (Fluxion Biosciences, Inc.). Bright-field and epifluorescence images were calibrated to 0.317712 μM/pixel. For bright-field images, a threshold was set using the Threshold tool and Slider tool to include all cells within each image while excluding any background area. The total percentage of area covered within this threshold was designated as the percentage of biofilm coverage, and these values were plotted over time. For epifluorescence images, the threshold was set to include all light areas (which were considered to be fluorescing cells), and the total percentage of the area covered within this threshold was designated as the percentage of fluorescing cells. These values were also plotted over time. All time points were plotted in 15-min intervals using GraphPad Prism software (GraphPad Software, La Jolla, CA).
