# Supplementary material for: Identification of Extracellular DNA-Binding Proteins in the Biofilm Matrix
Source: mBio. 2019 Jun 25;10(3):e01137-19. doi: 10.1128/mBio.01137-19 (PMC6593408; doi:10.1128/mBio.01137-19)
Supplement: TABLE S1 [file mBio.01137-19-st001.docx]

**Supplemental Table 1 comprehensive list of hits from SW screen**

| Membrane fraction | | | | | | | | |  |  |  |  |
| --- | --- | --- | --- | --- | --- | --- | --- | --- | --- | --- | --- | --- |
| Band # | ORF | Name | Location | Theoretical MW (KD) | pI | Percent Coverage | Quantitative Value | Evidence for DNA binding or comments | ^1^Foulston et al. (33) | ^2^Graf et al. <ECM> rank | ^3^Graf et al.  FC ECM/P | ^4^Graf et al.  FC ECM/FT |
| 1 | SAUSA300_1917 | Eap, extracellular adherence protein | extracellular membrane-associated | 65.7 | 9.9 | 50 | 238 | SW of KO mutant Figure 2a and eDNA release Figure X | no | 10 | 6.28 | 27.56 |
| 1 | SAUSA300_0955 | AtlA amidase domain | equatorial ring of cell surface | 60.8 | 9.6 | 68 | 179 |  | no | 1 | 1.22 | 3.94 |
| 1 | SAUSA300_1684 | hypothetical | membrane protein | 43.9 | 9.5 | 48 | 155 | fibrinogen like helix low confidence | no | 287 | 0.78 | 2.17 |
| *1* | *SAUSA300_1193* | *aerobic glycerol-3-phosphate dehydrogenase* | *Cytoplasmic/ inner membrane* | *62.4* | *7.0* | *42* | *154* |  | *yes* | *188* | *2.16* | *1.60* |
|  |  |  |  |  |  |  |  |  |  |  |  |  |
| 2 | SAUSA300_1533 | hypothetical | membrane protein | 35.2 | 5.2 | 57 | 225 |  | no | 92 | 1.40 | 3.52 |
| 2 | SAUSA300_0491 | cysteine synthase | cytoplasmic | 33.0 | 5.5 | 60 | 103 |  | no | nd | nd | Nd |
| 2 | SAUSA300_1790 | PrsA peptidyl-prolyl cis/trans isomerase | membrane lipoprotein | 35.6 | 9.0 | 38 | 100 | parvulin-like PPIase fold binds DNA, essential | no | 90 | 0.43 | 1.04 |
| 2 | SAUSA300_1729 | hypothetical | membrane protein | 35.0 | 6.5 | 68 | 96 | platelet integren β3 like helix high confidence | no | 443 | 1.04 | 1.05 |
| 2 | SAUSA300_0618 | ABC transporter, Mn/Zn transporter substrate-binding protein | membrane protein | 34.7 | 8.7 | 44 | 417 |  | no | 177 | 0.84 | 1.80 |
| 2 | SAUSA300_2201 | RplB, 50S ribosomal protein L2 | cytoplasmic/ribosome | 30.1 | 10.8 | 55 | 202 | binds 16S rRNA in the 70S ribosome, essential | no | 37 | 9.10 | 6.59 |
| 2 | SAUSA300_0753 | hypothetical | cytoplasmic | 34.2 | 9.3 | 58 | 120 | Epimerase | no | 907 | 2.26 | Nd |
| 2 | SAUSA300_0419 | lipoprotein | membrane | 31.4 | 8.8 | 51 | 75 | part of lpl cluster SAUSA300_0410-SAUSA300_0419 | no | 433 | 0.78 | 1.05 |
|  |  |  |  |  |  |  |  |  |  |  |  |  |
| *3* | *SAUSA300_2201* | *RplA, 50S ribosomal protein L1* | *cytoplasmic/ribosome* | *30.1* | *10.7* | *44* | *215* | *binds 23S rRNA, essential* | *yes* | *30* | *2.51* | *2.60* |
| *3* | *SAUSA300_2198* | *RpsC, 30S ribosomal protein S3* | *cytoplasmic/ribosome* | *24.1* | *9.8* | *67* | *89* | *binds mRNA in the 70S ribosome, essential* | *yes* | *34* | *1.75* | *1.93* |
| 3 | SAUSA300_1762 | EpiF, lantibiotic epidermin immunity protein F |  | 25.9 | 6.8 | 25 | 58 | ATP binding | no | nd | nd | nd |
| 3 | SAUSA300_0736 | YfiA ribosomal subunit interface protein | cytoplasmic | 22.2 | 5.3 | 35 | 54 |  | no | nd | nd | nd |
| *3* | *SAUSA300_2198* | *RpsC, 30S ribosomal protein S3* | *cytoplasmic/ribosome* | *24.3* | *9.8* | *35* | *309* | *binds mRNA in the 70S ribosome, essential* | *yes* | *34* | *1.75* | *1.93* |
| 3 | SAUSA300_1666 | RpsD, 30S ribosomal protein S4 | cytoplasmic/ribosome | 23.0 | 10.2 | 19 | 242 | binds 16S rRNA, essential | no | 35 | 2.24 | 4.17 |
| 3 | SAUSA300_2066 | uracil phosphoribosyltransferase | cytoplasmic | 23.0 | 10.2 | 47 | 98 |  | no | 177 | 0.84 | 1.80 |
|  |  |  |  |  |  |  |  |  |  |  |  |  |
| 4 | SAUSA300_2144 | hypothetical | membrane | 21.2 | 9.6 | 35 | 347 | C-terminal half similar to PrgH from Salmonella T3SS needle complex, high confidence | no | 390 | 0.58 | 3.78 |
| 4 | SAUSA300_1656 | Putative universal stress protein | cytoplasmic | 18.5 | 5.88 | 67 | 245 |  | no | 78 | 0.31 | 0.32 |
| 4 | SAUSA300_2142 | Asp23 | cytoplasmic | 20.3 | 8.2 | 70 | 195 |  |  |  |  |  |
| *4* | *SAUSA300_2187* | *RpsE, 30S ribosomal protein S5* | *cytoplasmic/ribosome* | *17.7* | *9.9* | *66* | *151* | *binds mRNA in the 70S ribosome, essential* | *yes* | *6* | *2.99* | *1.42* |
| 4 | SAUSA300_1685 | hypothetical lipoprotein | membrane | 18.0 | 7.5 | 58 | 107 | Four helix bundle serine2 chemotaxis receptor | no | 86 | 0.72 | 2.48 |
| 4 | SAUSA300_0079 | copper binding protein CopL | membrane | 20.1 | 9.0 | 46 | 61 | YdhK C-terminal domain from B subtilis, high confidence | no | nd | nd | nd |
|  |  |  |  |  |  |  |  |  |  |  |  |  |
| 5 | SAUSA300_1652 | hypothetical | cytoplasmic | 16.0 | 9.7 | 65 | 162, 178 | Adenine nucleotide alpha hydrolase-like domain (ANHH-like), high confidence | no | 52 | 0.94 | 1.45 |
| 5 | SAUSA300_1603 | RplU, 50S ribosomal protein L21 | cytoplasmic/ribosome | 11.3 | 9.9 | 72 | 139, 150 | binds 23S rRNA, essential | no | 24 | 7.69 | 3.73 |
| 5 | SAUSA300_2187 | RpsM, 30S ribosomal protein S13 | cytoplasmic/ribosome | 13.7 | 10.4 | 67 | 102, 115 | binds 16S rRNA, In 70S ribosome binds 23S rRNA and protein L5, contacts tRNA in A- and P-sites, essential | no | 25 | 4.34 | 5.31 |
| 5 | SAUSA300_1134 | RplS, 50S ribosomal protein L19 | cytoplasmic/ribosome | 13.4 | 11.5 | 54 | 88, 97 | 30S-50S interface, maybe aminoacyl-tRNA binding, essential | no | 26 | 4.54 | 2.21 |
| 5 | SAUSA300_2190 | RpsH, 30S ribosomal protein S8 | cytoplasmic/ribosome | 14.8 | 9.3 | 52 | 71, 73 | binds 16S rRNA, essential | no | 70 | 0.74 | 0.55 |
| 5 | SAUSA300_0522 | RplK, 50S ribosomal protein L11 | cytoplasmic/ribosome | 14.9 | 9.0 | 36 | 69, 78 | part of ribosomal stalk, essential | no | 59 | 1.72 | 1.02 |
| 5 | SAUSA300_2171 | RpsI, 30S ribosomal protein S9 | cytoplasmic/ribosome | 14.8 | 10.6 | 35 | 66, 62 | essential | no | 36 | 6.40 | 4.68 |
| 5 | SAUSA300_2177 | RplQ, 50S ribosomal protein L17 | cytoplasmic/ribosome | 13.7 | 9.8 | 40 | 59, 58 | structural constituent of ribosome, essential | no | 68 | 1.49 | 0.63 |
| 5 | SAUSA300_2179 | RpsK, 30S ribosomal protein S11 | cytoplasmic/ribosome | 13.8 | 11.2 | 36 | 58, 64 | binds 16S rRNA, essential | no | 67 | 6.31 | 3.25 |
| 5 | SAUSA300_2199 | RplV, 50S ribosomal protein L22 | cytoplasmic/ribosome | 12.8 | 9.9 | 68 | 52, 36 | binds 23S rRNA, essential | no | 18 | 6.35 | 3.54 |
| 5 | SAUSA300_0605 | SarA | cytoplasmic | 14.7 | 8.1 | 33 | 44, 52 | DNA binding transcriptional regulator | no | 19 | 1.68 | 3.62 |
| 5 | SAUSA300_1625 | RplT, 50S ribosomal protein L20 | cytoplasmic/ribosome | 13.7 | 11.3 | 31 | 42, 37 | binds 23S rRNA, essential | no | 42 | 4.52 | 4.08 |
| 5 | SAUSA300_0067 | universal stress protein family | cytoplasmic | 16.9 | 6.0 | 43 | 35, 34 |  | no | 52 | 0.95 | 1.45 |
| 5 | SAUSA300_1788 | universal stress protein family | cytoplasmic | 13.2 | 6.1 | 43 | 25, 32 |  | no | 519 | 0.33 | 0.240 |
| 5 | SAUSA300_2573 | IsaB | cytoplasmic | 19.4 | 9.7 | 35 | 31,27 |  | no | 28 | 0.71 | 4.18 |
|  |  |  |  |  |  |  |  |  | no |  |  |  |
| 5 | SAUSA300_2172 | RplM, 50S ribosomal protein L13 | cytoplasmic/ribosome | 16.3 | 9.3 | 86 | 170 | binds 23S rRNA, essential | no | 38 | 1.27 | 0.73 |
| 5 | SAUSA300_5031 | RpsG, 30S ribosomal protein S7 | cytoplasmic/ribosome | 14.4 | 9.6 | 58 | 121 | binds 16S rRNA, essential | no | 21 | 6.12 | 4.36 |
| 5 | SAUSA300_0693 | SaeP | lipoprotein | 16.0 | 9.1 | 47 | 96 |  | no | 65 | 1.23 | 1.14 |
| 5 | SAUSA300_2185 | RplO, 50S ribosomal protein L15 | cytoplasmic/ribosome | 15.6 | 10.3 | 63 | 94 |  | no | 61 | 4.71 | 4.34 |
| 5 | SAUSA300_2197 | RplP, 50S ribosomal protein L16 | cytoplasmic/ribosome | 16.2 | 10.6 | 44 | 84 | binds 23S rRNA, A site tRNA, maybe T site tRNA, essential | no | 40 | 15.70 | 6.46 |
| 5 | SAUSA300_0067 | universal stress protein family | cytoplasmic | 16.9 | 6.0 | 43 | 65 |  | no | 52 | 0.95 | 1.45 |
| 5 | SAUSA300_0015 | RplI, 50S ribosomal protein L9 | cytoplasmic/ribosome | 16.5 | 9.4 | 72 | 60 | Binds 23S rRNA, A site tRNA, maybe T site tRNA, not essential | no | 333 | 1.24 | 0.50 |
| 5 | SAUSA300_2573 | IsaB | cytoplasmic | 19.4 | 9.7 | 35 | 41 |  | no | 28 | 0.71 | 4.18 |
| 5 | SAUSA300_1698 | hypothetical | cytoplasmic | 15.7 | 9.2 | 54 | 37 | Very low homology to human alpha-synuclein | no | 417 | 0.44 | 0.66 |
| Spent media | | | | | | | | |  |  |  |  |
| Band # | ORF | Name | Location | Theoretical MW (KD) | pI | Percent Coverage | Quantitative Value | Evidence for DNA binding or comments |  |  |  |  |
| *6* |  | *Glycerol kinase* | *cytoplasmic* |  |  |  | *88* |  | *yes* | *617* | *1.30* | *0.68* |
|  |  |  |  |  |  |  |  |  |  |  |  |  |
| 7 | SAUSA300_0955 | AtlA amidase and glucosaminidase domains | equatorial ring of cell surface | 116.5 | 9.6 | 67 | 1270 |  | no | 1 | 1.22 | 3.94 |
|  |  |  |  |  |  |  |  |  |  |  |  |  |
| 8 | SAUSA300_0320 | Geh, triacylglycerol lipase | secreted | 72.2 | 9.0 | 52 | 932 |  | no | 8 | 0.79 | 0.62 |
| 8 | SAUSA300_0955 | AtlA amidase domain | equatorial ring of cell surface | 60.8 | 9.6 | 30 | 240 |  | no | 1 | 1.22 | 3.94 |
| 8 | SAUSA300_2579 | N-acetylmuramoyl-L-alanine amidase | equatorial ring of cell surface | 69.2 | 6.4 | 23 | 98 |  | no | 45 | 0.30 | 0.59 |
|  |  |  |  |  |  |  |  |  |  |  |  |  |
| 9 | SAUSA300_0703 | LtaS, SpsB-liberated domain | equatorial ring of cell surface | 49.3 | 8.6 | 55 | 799 |  | no | 55 | 0.16 | 0.55 |
| 9 | SAUSA300_0955 | AtlA glucosaminidase domain | equatorial ring of cell surface | 54.4 | 9.7 | 55 | 430 |  | no | 1 | 1.22 | 3.94 |
|  |  |  |  |  |  |  |  |  |  |  |  |  |
| 10 | SAUSA300_1058 | Hla, alpha-toxin | secreted | 36.0 | 8.7 | 67 | 383 |  | no | 83 | 0.81 | 0.26 |
| 10 | SAUSA300_0320 | Geh, triacylglycerol lipase | secreted | 42 | 9.6 | 23 | 140 |  | no | 8 | 0.79 | 0.62 |
| 10 | SAUSA300_0491 | CysK, cysteine synthase | cytoplasmic | 33.0 | 5.5 | 60 | 115 |  | no | 448 | 0.93 | 0.63 |
| 10 | SAUSA300_1382 | LukS, PVL toxin | secreted | 35.7 | 9.2 | 50 | 112 |  | no | 11 | 2.47 | 0.84 |
| 10 | SAUSA300_0681 | Fe/Zn/Cu ABC transporter substrate binding protein | secreted | 34.7 | 8.7 | 61 | 92 |  | no | 383 | 0.10 | 1.17 |
| 10 | SAUSA300_0536 | Hsp31, chaperone | secreted | 32.2 | 5.0 | 37 | 83 |  | no |  |  |  |
| 10 | SAUSA300_1790 | PrsA peptidyl-prolyl cis/trans isomerase | membrane lipoprotein | 35.6 | 9.0 | 44 | 76 | parvulin-like PPIase fold binds DNA, essential | no | 90 | 0.43 | 1.04 |
| 10 | SAUSA300_1150 | Elongation factor Ts | cytoplasmic | 32.5 | 5.1 | 55 | 56 | Binds elongation factor Tu-GDP complex | no | 29 | 0.89 | 0.66 |
| 10 | SAUSA300_0479 | RplY, 50S ribosomal protein L25 | cytoplasmic/ribosome | 23.8 | 4.4 | 35 | 36 | binds 5S rRNA, | no | 63 | 0.94 | 0.71 |
| *10* | *SAUSA300_0553* | *Elongation factor Tu* | *cytoplasmic* | *43.1* | *4.5* | *27* | *36* | *binds elongation factor Ts-GDP complex* | *yes* | *2* | 1.41 | 0.83 |
| *10* | *SAUSA300_0235* | *Lactate dehydrogenase* | *cytoplasmic* | *29.4* | *5.1* | *28* | *30* |  | *yes* | *159* | *31.26* | *0.37* |
|  |  |  |  |  |  |  |  |  |  |  |  |  |
| 11 | SAUSA300_1988 | δ-toxin | secreted | 3.0 | 8.7 | 85 | 184 |  | no | 7 | 1.04 | 0.41 |
| 11 | SAUSA300_ | PSMα4 | secreted | 2.2 | 9.7 | 85 | 167 |  | no | 9 | 4.53 | 3.86 |
| 11 | SAUSA300_1067 | PSMβ1 | secreted | 4.5 | 4.9 | 91 | 134 |  | no | 71 | 0.76 | 0.26 |
| 11 | SAUSA300_ | PSMα1 | secreted | 2.3 | 9.7 | 86 | 84 |  | no | nd | nd | nd |
|  |  |  |  |  |  |  |  |  |  |  |  |  |

^1^Protein detected in the biofilm matrix by Poulston et al. Yes indicates protein was detected and no indicates protein was not detected

^2^Rank order of average Intensity-based Absolute Quantification for proteins detected in the biofilm extracellular matrix by Graf et al. nd indicates not detected by Graf et al.

^3^Fold-change comparing protein abundance in biofilm extracellular matrix with protein abundance in supernatant form planktonic cells as reported by Graf et al.

^4^Fold-change comparing protein abundance in biofilm extracellular matrix with protein abundance in biofilm flow-through as reported by Graf et al.
