# Supplementary figures and images for: Identification of Extracellular DNA-Binding Proteins in the Biofilm Matrix
Source: mBio. 2019 Jun 25;10(3):e01137-19. doi: 10.1128/mBio.01137-19 (PMC6593408; doi:10.1128/mBio.01137-19)

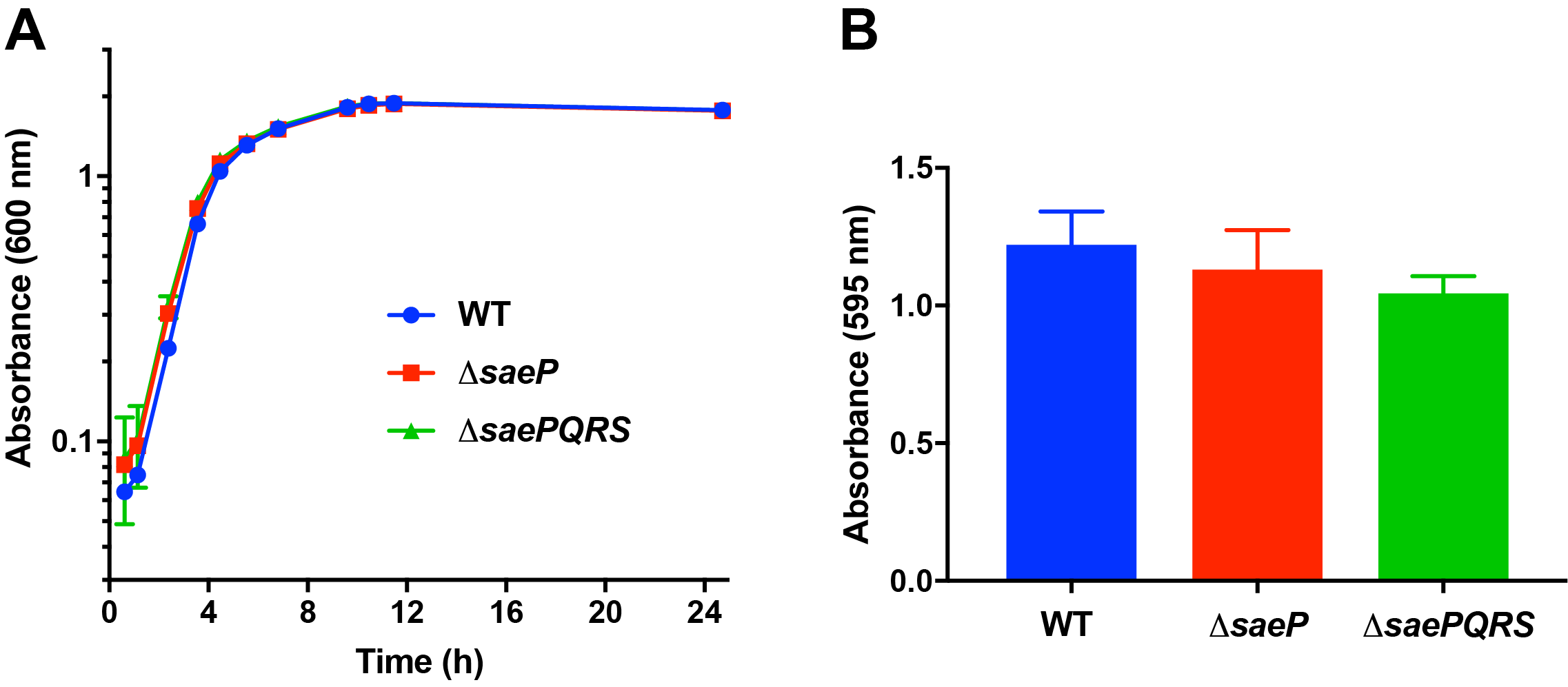

Supplement: FIG S1 [file mBio.01137-19-sf001.tif]

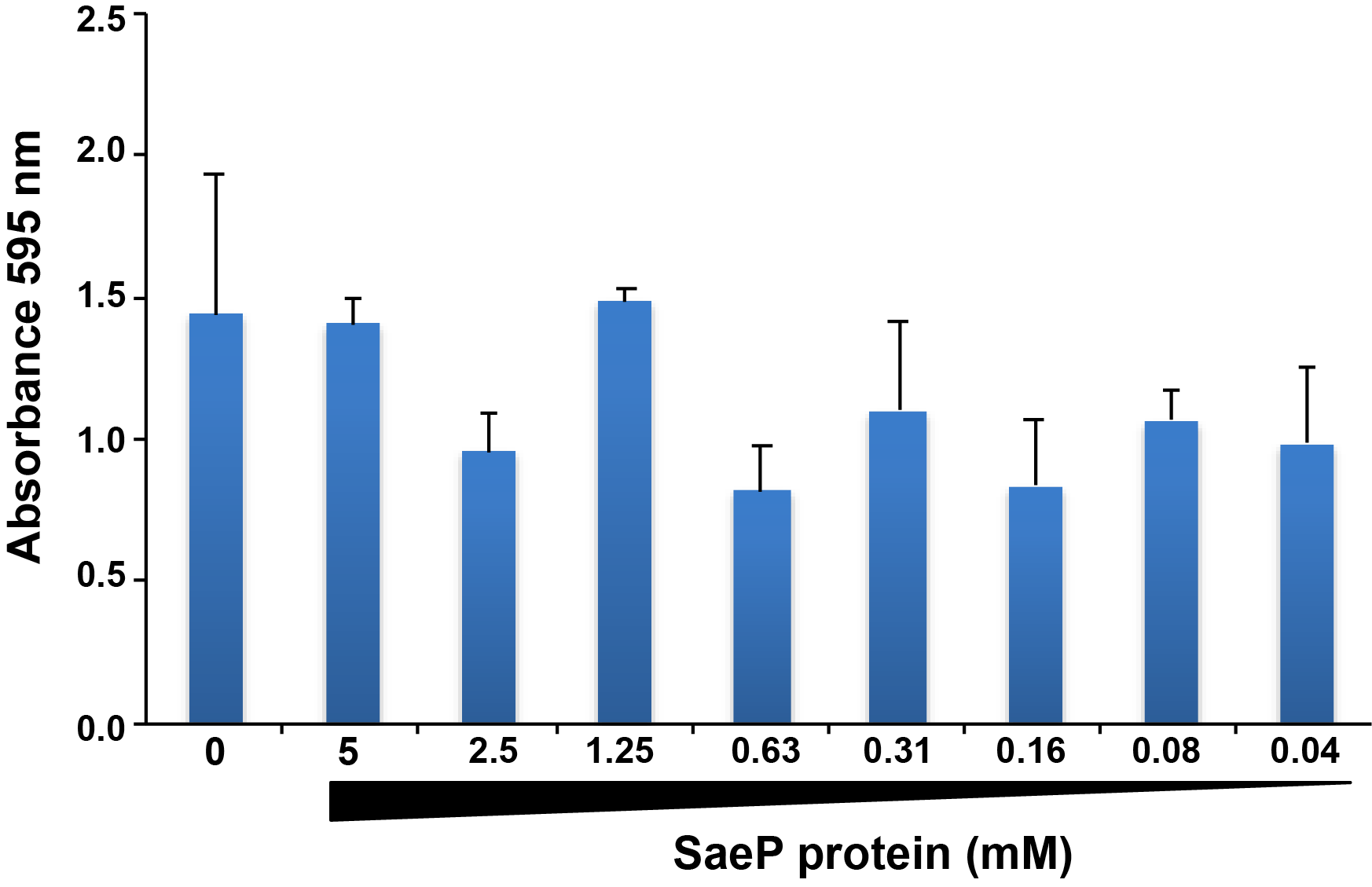

Supplement: FIG S2 [file mBio.01137-19-sf002.tif]

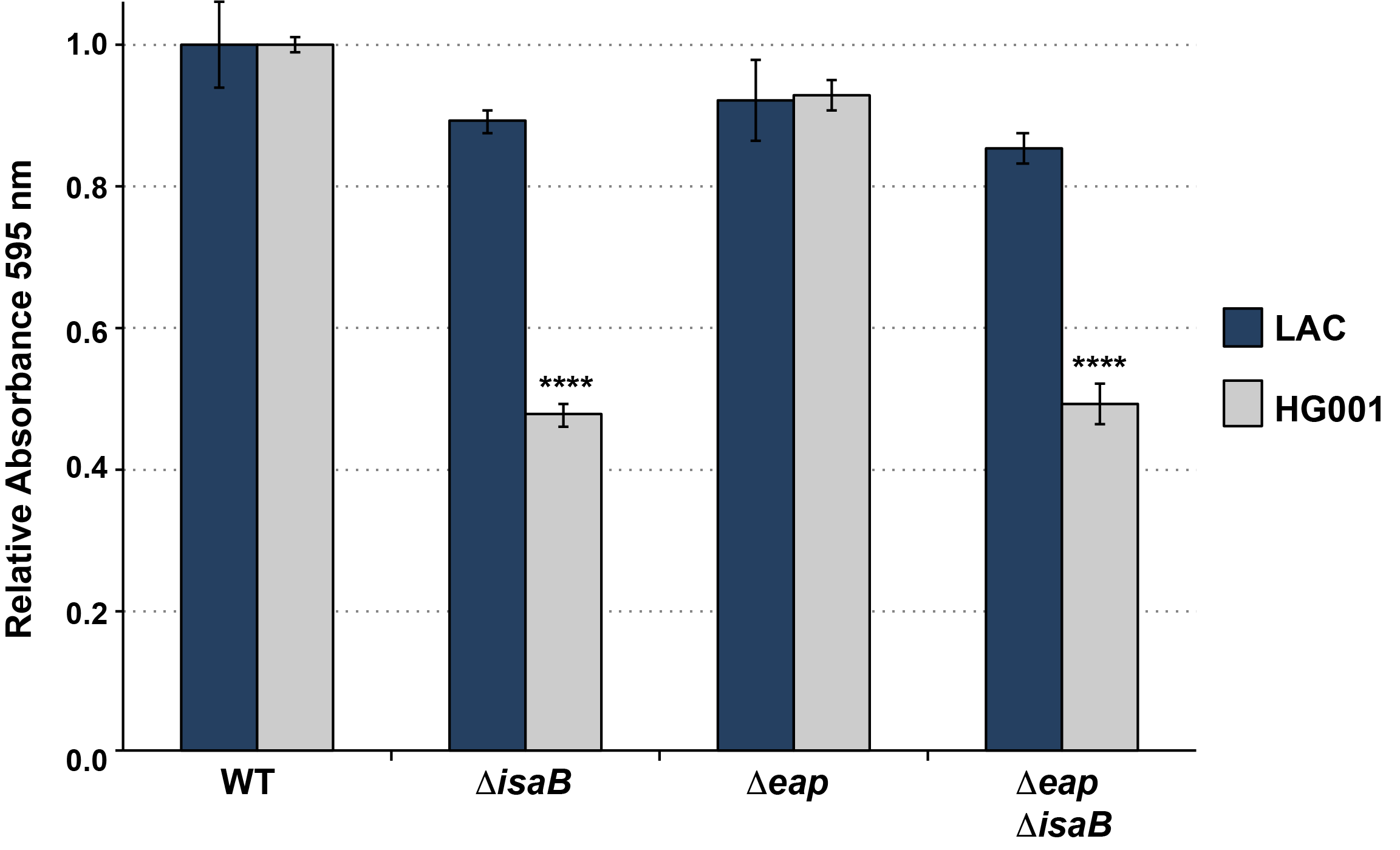

Supplement: FIG S4 [file mBio.01137-19-sf004.tif]

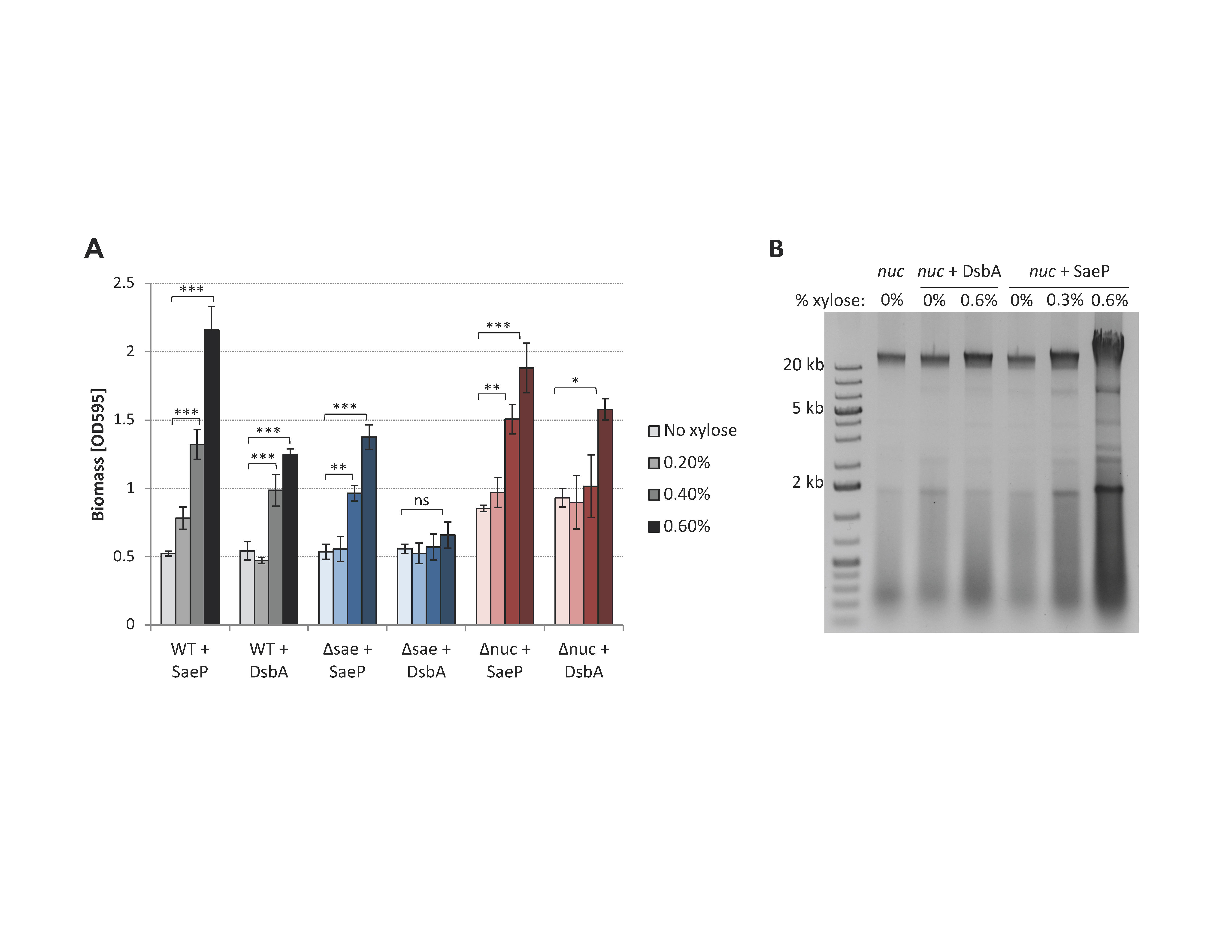

Supplement: FIG S3 [file mBio.01137-19-sf003.jpg]

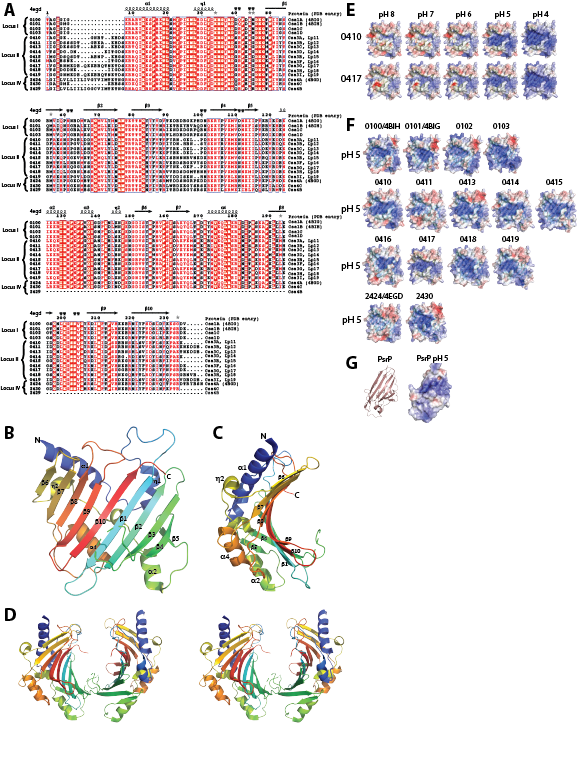

Supplement: FIG S5 [file mBio.01137-19-sf005.tif]

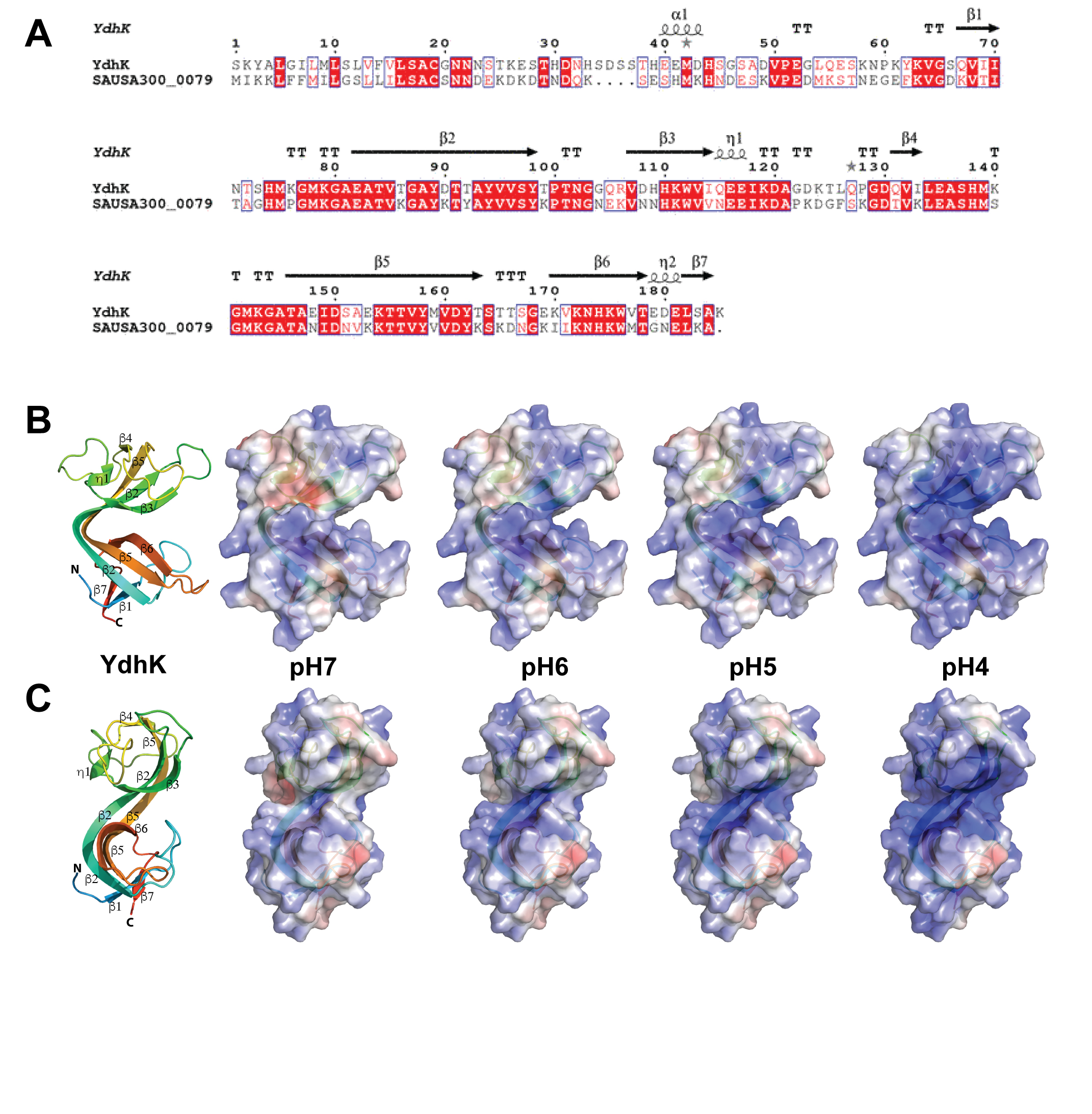

Supplement: FIG S6 [file mBio.01137-19-sf006.jpg]
