# Supplementary material for: Identification of Extracellular DNA-Binding Proteins in the Biofilm Matrix
Source: mBio. 2019 Jun 25;10(3):e01137-19. doi: 10.1128/mBio.01137-19 (PMC6593408; doi:10.1128/mBio.01137-19)
Supplement: TABLE S2 [file mBio.01137-19-st002.docx]

**Supplemental Table 2.** Primer and plasmid list

| **Primer** | **Sequence 5' to 3'** | **Description** |
| --- | --- | --- |
| pSKerm for | TTAGCTTCCTTAGCTCCTGAAAATCTCGTC | Plasmid sequencing primers |
| pSKerm rev | CAAATCCGCCGGGAGCGGATTTGAACGTT | Plasmid sequencing primers |
| pEPSA5for2 | AGTTATAAAATAGATATCTCGGACCGTCAT | 100 bp upstream of MCS |
| pEPSA5rev | GGCAAATTCTGTTTTATCAGACCG | 100 bp downstream of MCS |
| 00793 | GTTGTTGGATCCTTATCGTGCTTTCAGCTCATTTCC | SAUAS300_0079 forward |
| 00795 | GTTGTTGCTAGCTCCAATAATGATGAAAAAGATAAAGACAC | SAUAS300_0079 reverse |
| 01003 | GTTGTTGGATCCTTATACATCTCCGCTTTTTTTATAATCTAAGC | SAUSA300_0100 forward |
| 01005 | GTTGTTGCTAGCGGCATAGGTAAAGAAGCGGAAG | SAUSA300_0100 reverse |
| 23543 | GTTGTTGGATCCCTATTTGATTTTATCTTTTAATAACTTCTC | *dsbA* forward |
| 23545 | GTTGTTGCTAGCGGTAAAAAAGAATCAGCAACGAC | *dsbA* reverse |
| CBR1 | GTTGGTACCGACGATTGACTGCCACCACCG | *isbA* forward, KpnI |
| CBR2 | AAGTTACGCGTGTTGTTCTCGAGCTCTCTTTTCCATACAAATATTTTATTCC | *isbA* reverse, MluI-XhoI |
| CBR3 | GTATGGAAAAGAGAGCTCGAGAACAACACGCGTGGCATATTAAGGCTAGAGTGTG | *isbA* forward, MluI-XhoI |
| CBR4 | AAGAATTCCGCATCACTCTTTTGAGTCACC | *isbA* reverse, EcoRI |
| CBR5 | GTTGTTGGTACCGACGATTGACTGCCACCACCG | *isbA* forward, KpnI |
| CBR6 | GTTGTTGAATTCCGCATCACTCTTTTGAGTCACC | *isbA* reverse, EcoRI |
| CEF16 | GTTGTTAAGCTTTTATTGTGGCAAAAGGTTT | 300bp upstream of *saeP*, HindIII |
| CEF17 | GTTGTTCCCGGGATTATTAGGCGGCATACAG | 235bp downstream of *saeS*, XmaI |
| CEF27 | GAAGTAACAATCTATGCTAAAAAAGCTCACGTCATC | mutating saeP Cys133 to Ala, forward |
| CEF28 | GATGACGTGAGCTTTTTTAGCATAGATTGTTACTTC | mutating saeP Cys133 to Ala, reverse |
| CEF43 | GTTGTTGCTAGCGGTAATTCTAATTCACAAGATCAAGG | NheI, immediately after Cys in signal sequence of *saeP* |
| CEF44 | GTTGTTGGATCCTTATTTTAATTTAGCGCCGCCGAA | directly downstream of *saeP*, stop codon, BamHI; revcomp |
| CEF64 | GTTGTTGAATTCTTAGGAGGATGATTATTTATGAATACAAAATATTTTTTAGCAGCTGG | EcoRI, SOD RBS, *saeP* fwd |
| CEF65 | GTTGTTCTCGAGTTAACCCATTTGCTGTCCACCAGTCATGCTAGCCATAGAGCCACCTTTTAATTTAGCGCCGCCGAA | XhoI, downstream of *saeP*, C-term T7 tag with Gly-Gly-Ser linker Revcomp |
| CEF217 | GTTGTTGAATTCTTAGGAGGATGATTATTTATGACTAAAAAATTACTAACATTATTTATAGTGAGC | EcoRI, Sod RBS, *dsbA*, fwd |
| CEF218 | GTTGTTCTCGAGTTAACCCATTTGCTGTCCACCAGTCATGCTAGCCATAGAGCCACCTTTGATTTTATCTTTTAATAACTTCTC | XhoI, downstream of *dsbA*, C-term T7 tag with Gly-Gly-Ser linker Revcomp |
| CEF226 | GCTTTAGGTGCTGCTGGTAATTCTAAT | mutating SaeS C21A, fwd |
| CEF227 | ATTAGAATTACCAGCAGCACCTAAAGC | mutating SaeS C21A, rev |
| CEF232 | GTTGTTGAATTCTTAGGAGGATGATTATTT | SAUSA300_0175, forward |
| CEF233 | GTTGTTCTCGAGTTAACCCATTTGCTGTCCACCAGTCATGCTAGCCATAGAGCCACCTGAACGCGATGCCTCC | SAUSA300_0175 rev, c-term T7 |
| CEF234 | GTTGTTGAATTCTTAGGAGGATGATTATTT | SAUSA300_1436, fwd |
| CEF235 | GTTGTTCTCGAGTTAACCCATTTGCTGTCCACCAGTCATGCTAGCCATAGAGCCACCGTTAATATTGGCTCCTGGTACTGC | SAUSA300_1436 rev, c-term T7 |
| CEF236 | GTTGTTGAATTCTTAGGAGGATGATTATTT | SAUSA300_1478, fwd |
| CEF237 | GTTGTTCTCGAGTTAACCCATTTGCTGTCCACCAGTCATGCTAGCCATAGAGCCACCTTTTTTCATATTATTTTCTTCATAAACTGG | SAUSA300_1478 rev, c-term T7 |
| MO122 | CGTTGATATTTGTTGATAGTC | Olson, 2013 IRD700 probe |
| MO123 | GTTCATTATGAAGTCCCTCC | Olson, 2013 IRD700 probe |
| JL14 | CATTGTTTTTTGTGTACTTCAATTACTATAACA | Confirmation of *isaB* mutant |
| JL15 | CGATTATTGCGTCTTACATAGTTGTTTG | Confirmation of *isaB* mutant |
| JL6 | GAATGTTGTTATGACATTTAAGTTTGAAGCTTGG | confirmation of *eap*::erm mutant |
| JL7 | GTAGAGATGTGATGCTTGAATGTTTGAAGTG | confirmation of *eap*::erm mutant |
| JL34 | CTCAGCAAATGCATCACAAAC | confirmation of *nuc* mutant |
| JL35 | TCAATTTTCTTTGCATTTTCTACCA | confirmation of *nuc* mutant |
| lpl_up_fw | GCAAATGCAGTTGTGAAGGT | construction of Δcsa mutants |
| lpl_up_rv_XhoI | ggggctcgagCGCTTCACGTCTCTCTGTTTT | construction of Δcsa mutants |
| lpl_dw_fw_XhoI | ggggctcgagTGGACCGTCTGAAGGTGAG | construction of Δcsa mutants |
| lpl_dw_rv_KpnI | ccccggtaccGACTGCGCAATTGTTAAAGC | construction of Δcsa mutants |
| LocusI_up_fw_EcoRI | ccccgaattcTGAGTGGTTGGTATCATTCAGC | construction of Δcsa mutants |
| LocusI_up_rv_XhoI | ggggctcgagAAAAATTTCACAAACAATCTGTTCA | construction of Δcsa mutants |
| LocusI_dw_fw_XhoI | ggggctcgagTCCACACACACGATTCAATATG | construction of Δcsa mutants |
| LocusI_dw_rv_KpnI | ccccggtaccATGGCGCATTGAGCTTTTT | construction of Δcsa mutants |
| 2430_up_fw_EcoRI | ccccgaattcGGCAAATAGCACCTGGATTG | construction of Δcsa mutants |
| 2430_up_rv_KpnI | ccccggtaccCCAGAGCCTCAACCTTTTTG | construction of Δcsa mutants |
| 2429_dw_fw_KpnI | ccccggtaccCAAAGCCTATACCGGATGACA | construction of Δcsa mutants |
| 2429_dw_rv_Sal | ccccgtcgaCGTCTTGGTAGCCTTTAACTTTG | construction of Δcsa mutants |
| 2424_up_fw_SalI | ccccgtcgacTCCCAAAAGAAAAATTGATGA | construction of Δcsa mutants |
| 2424_up_rv_KpnI | ccccggtaccTAATCGCCATCACCTTCATA | construction of Δcsa mutants |
| 2424_dw_fw_KpnI | ccccggtaccTCGATATCTAAAGTGAGCTGTGA | construction of Δcsa mutants |
| 2424_dw_rv_EcoRI | ccccgaattcAAGAACCGCATTTGATTTTC | construction of Δcsa mutants |
| Lpl_up_seq1 | CAAAATTTCCACTTAATAATTCACC | Δcsa mutant sequencing primers |
| Lpl_up_seq2 | ATTAGATAAGAATGCTGACCAAGC | Δcsa mutant sequencing primers |
| Lpl_up_seq3 | AGCTTTGATGTAGATCATG | Δcsa mutant sequencing primers |
| Lpl_dw_seq1 | TGACGCTTTCGAAATAGCACT | Δcsa mutant sequencing primers |
| Lpl_dw_seq2 | CCGCATCTCTTCCACCTAGT | Δcsa mutant sequencing primers |
| Lpl_dw_seq3 | TTGCAGAAAATGATGCATTGA | Δcsa mutant sequencing primers |
| locusI_up_seq | CGTGTCAGCACCATAACCAC | Δcsa mutant sequencing primers |
| locusI_dwn_seq | TGTACGCGGGACATAATGAA | Δcsa mutant sequencing primers |
| lpl_up_fw | GCAAATGCAGTTGTGAAGGT | Δcsa mutant sequencing primers |
| 2429-30_seq1 | AACGATGGATTTAGCACTGGA | Δcsa mutant sequencing primers |
| 2429-30_seq2 | GATCCTACGGATGAGCCTTTT | Δcsa mutant sequencing primers |
| 2429-30_seq3 | TTGATATTATTACCGGACATTCG | Δcsa mutant sequencing primers |
| 2424_seq1 | CAAACGCTCAGCATCAATGT | Δcsa mutant sequencing primers |
| 2424_seq2 | AGTGAGGTTATTGCGCCTGA | Δcsa mutant sequencing primers |
| lpl_chrom_colony_up | CATGTAGATGAAATTTACGGAAGC | Δcsa mutant confirmation |
| lpl_chrom_colony_dwn | TGATGCAAGCACTTGATCTTT | Δcsa mutant confirmation |
| locusI_chrom_col_up | TCCTAAGGGTTTTTAATCATATGTC | Δcsa mutant confirmation |
| locusI_chrom_col_dwn | GATCCAAATGCATGGTCAAA | Δcsa mutant confirmation |
| 2429-30_chrom_col_up | TGGGACACATGCCGTTACTA | Δcsa mutant confirmation |
| 2429-30_chrom_col_dwn | TCCAAATTTATGTGCCATCG | Δcsa mutant confirmation |
| 2424_chrom_col_up | TAAGAAAAGGTGGCGCGTAT | Δcsa mutant confirmation |
| 2424_chrom_col_dwn | GCGTGTTTTAGCATTAAGAGCA | Δcsa mutant confirmation |
